# Abortion Surveillance — United States, 2014

**DOI:** 10.15585/mmwr.ss6625a1

**Published:** 2018-11-23

**Authors:** Tara C. Jatlaoui, Jill Shah, Michele G. Mandel, Jamie W. Krashin, Danielle B. Suchdev, Denise J. Jamieson, Karen Pazol

**Affiliations:** 1 Division of Reproductive Health, National Center for Chronic Disease Prevention and Health Promotion, CDC; 2 Oak Ridge Institute for Science and Education (ORISE) Fellow

## Abstract

**Problem/Condition:**

Since 1969, CDC has conducted abortion surveillance to document the number and characteristics of women obtaining legal induced abortions in the United States.

**Period Covered:**

2014.

**Description of System:**

Each year, CDC requests abortion data from the central health agencies of 52 reporting areas (the 50 states, the District of Columbia, and New York City). The reporting areas provide this information voluntarily. For 2014, data were received from 49 reporting areas. For trend analysis, abortion data were evaluated from 48 areas that reported data every year during 2005–2014. Census and natality data, respectively, were used to calculate abortion rates (number of abortions per 1,000 women aged 15–44 years) and ratios (number of abortions per 1,000 live births).

**Results:**

A total of 652,639 abortions were reported to CDC for 2014. Of these abortions, 98.4% were from the 48 reporting areas that provided data every year during 2005–2014. Among these 48 reporting areas, the abortion rate for 2014 was 12.1 abortions per 1,000 women aged 15–44 years, and the abortion ratio was 193 abortions per 1,000 live births. From 2013 to 2014, the total number and rate of reported abortions decreased 2%, and the ratio decreased 3%. From 2005 to 2014, the total number, rate, and ratio of reported abortions decreased 21%, 22%, and 18%, respectively. In 2014, all three measures reached their lowest level for the entire period of analysis (2005–2014).

In 2014 and throughout the period of analysis, women in their 20s accounted for the majority of abortions and had the highest abortion rates; women in their 30s and older accounted for a much smaller percentage of abortions and had lower abortion rates. In 2014, women aged 20–24 and 25–29 years accounted for 32.2% and 26.7% of all reported abortions, respectively, and had abortion rates of 21.3 and 18.4 abortions per 1,000 women aged 20–24 and 25–29 years, respectively. In contrast, women aged 30–34, 35–39, and ≥40 years accounted for 17.1%, 9.7%, and 3.6% of all reported abortions, respectively, and had abortion rates of 11.9, 7.2, and 2.6 abortions per 1,000 women aged 30–34 years, 35–39 years, and ≥40 years, respectively. From 2005 to 2014, the abortion rate decreased among women aged 20–24, 25–29, 30–34, and 35–39 years by 27%, 16%, 12%, and 5%, respectively, but increased 4% among women aged ≥40 years.

In 2014, adolescents aged <15 and 15–19 years accounted for 0.3% and 10.4% of all reported abortions, respectively, and had abortion rates of 0.5 and 7.5 abortions per 1,000 adolescents aged <15 and 15–19 years, respectively. From 2005 to 2014, the percentage of abortions accounted for by adolescents aged 15–19 years decreased 38%, and their abortion rate decreased 49%. These decreases were greater than the decreases for women in any older age group.

In contrast to the percentage distribution of abortions and abortion rates by age, abortion ratios in 2014 and throughout the entire period of analysis were highest among adolescents and lowest among women aged 30–39 years. Abortion ratios decreased from 2005 to 2014 for women in all age groups.

In 2014, the majority (64.9%) of abortions were performed at ≤8 weeks’ gestation, and nearly all (91.0%) were performed at ≤13 weeks’ gestation. Few abortions were performed between 14 and 20 weeks’ gestation (7.7%) or at ≥21 weeks’ gestation (1.3%). During 2005–2014, the percentage of all abortions performed at ≤13 weeks’ gestation remained consistently high (≥90.9%). Among abortions performed at ≤13 weeks’ gestation, there was a shift toward earlier gestational ages, as the percentage performed at ≤6 weeks’ gestation increased 9%, and the percentage of all other gestational ages at ≤13 weeks’ gestation decreased 0%–12%.

In 2014, among reporting areas that included medical (nonsurgical) abortion on their reporting form, 22.5% of all abortions were performed by early medical abortion (a nonsurgical abortion at ≤8 weeks’ gestation), 66.9% were performed by surgical abortion at ≤13 weeks’ gestation, and 9.1% were performed by surgical abortion at >13 weeks’ gestation; all other methods were uncommon (<1.5%). Among abortions performed at ≤8 weeks’ gestation that were eligible for early medical abortion on the basis of gestational age, 33.3% were completed by this method.

In 2014, women with one or more previous live births accounted for 59.5% of abortions, and women with no previous live births accounted for 40.4%. Women with one or more previous induced abortions accounted for 44.9% of abortions, and women with no previous abortion accounted for 55.1%. Women with three or more previous births accounted for 13.8% of abortions, and women with three or more previous abortions accounted for 8.6% of abortions.

Deaths of women associated with complications from abortion for 2014 are being assessed as part of CDC’s Pregnancy Mortality Surveillance System. In 2013, the most recent year for which data were available, four women were identified to have died as a result of complications from legal induced abortion.

**Interpretation:**

Among the 48 areas that reported data every year during 2005–2014, the decreases in the total number, rate, and ratio of reported abortions that occurred during 2010–2013 continued from 2013 to 2014, resulting in historic lows for all three measures of abortion.

**Public Health Action:**

The data in this report can help program planners and policymakers identify groups of women with the highest rates of abortion. Unintended pregnancy is the major contributor to induced abortion. Increasing access to and use of effective contraception can reduce unintended pregnancies and further reduce the number of abortions performed in the United States.

## Introduction

This report summarizes abortion data for 2014 that were provided voluntarily to CDC by the central health agencies of 49 reporting areas (the District of Columbia [DC]; New York City; and 47 states, excluding California, Maryland, and New Hampshire). Data obtained every year during 2005–2014 from 48 reporting areas (excluding California, Louisiana, Maryland, and New Hampshire) were used for trend analyses. Since 1969, CDC has conducted abortion surveillance to document the number and characteristics of women obtaining legal induced abortions in the United States (*1*). Following nationwide legalization of abortion in 1973, the total number, rate (number of abortions per 1,000 women aged 15–44 years), and ratio (number of abortions per 1,000 live births) of reported abortions increased rapidly, reaching the highest levels in the 1980s before decreasing at a slow yet steady pace (*2*–*4*). During 2006–2008, a break occurred in the previously sustained pattern of decrease (*5*–*8*) although this break has been followed in all subsequent years by even greater decreases (*9*–*14*). Nonetheless, throughout the years, the incidence of abortion has varied considerably across subpopulations and remains higher in some demographic groups than others (*15*–*20*). Continued surveillance is needed to monitor long-term changes in the incidence of abortion in the United States.

## Methods

### Description of the Surveillance System

Each year, CDC requests tabulated data from the central health agencies of 52 reporting areas (the 50 states, the District of Columbia, and New York City) to document the number and characteristics of women obtaining legal induced abortions in the United States. For the purpose of surveillance, a legal induced abortion* is defined as an intervention performed within the limits of state law by a licensed clinician (e.g., a physician, nurse-midwife, nurse practitioner, or physician assistant) that is intended to terminate a suspected or known intrauterine pregnancy.

In most states, collection of abortion data is facilitated by the legal requirement for hospitals, facilities, and physicians to report all abortions to a central health agency although reporting is not complete in all areas with these requirements (*21*). Moreover, these central health agencies then voluntarily report the abortion data they have collected through their independent surveillance systems and provide only aggregate numbers to CDC (*22*). Although reporting to CDC is voluntary, most reporting areas provide their aggregate abortion numbers. During 2005–2014, a total of 48 reporting areas provided CDC a continuous annual record of abortion numbers^†^ and in 2014, CDC obtained aggregate abortion numbers from 49 reporting areas (excludes California, Maryland, and New Hampshire).

Although CDC obtains aggregate abortion numbers from most of the central health agencies, the level of detail received on the characteristics of women obtaining abortions varies considerably from year to year and by reporting area. To encourage more uniform collection of these details, CDC has collaborated with the National Association of Public Health Statistics and Information Systems to develop reporting standards and provide technical guidance for vital statistics personnel who collect and summarize abortion data within the United States. However, because the collection and reporting of abortion data are not federally mandated, many reporting areas have developed their own data collection forms and therefore do not collect or provide all the information or level of detail included in this report.

### Variables and Categorization of Data

Each year, CDC sends suggested templates to the central health agencies for compilation of abortion data in aggregate. Aggregate abortion numbers, without individual-level records, are requested for the following variables:

- Maternal age in years (<15, 15–19 by individual year, 20–24, 25–29, 30–34, 35–39, or ≥40)
- Gestational age in completed weeks at the time of abortion (≤6, 7–20 by individual week, or ≥21)
- Race (black, white, or other [including Asian, Pacific Islander, other races, and multiple races])
- Ethnicity (Hispanic or non-Hispanic)
- Method type (surgical abortion,^§^ intrauterine instillation, medical [nonsurgical] abortion, or hysterectomy/hysterotomy)
- Marital status (married [including currently married or separated] or unmarried [including never married, widowed, or divorced])
- Number of previous live births (0, 1, 2, 3, or ≥4)
- Number of previous abortions (0, 1, 2, or ≥3)
- Maternal residence (the state, reporting area, territory, or foreign country in which the woman obtaining the abortion lived; or, if additional details are unavailable, in-reporting area versus out-of-reporting area)

Beginning with 2014 data, some areas reported gestational age to CDC by probable postfertilization age or clinical estimate of gestation based on date of conception. To make these data consistent with gestational age data reported by clinician’s estimate (*23*), 2 weeks were added to postfertilization age to account for time after last menstrual period until ovulation in a standard 28-day cycle because fertilization occurs around the time of ovulation (*24*). No modifications were made to data reported as clinician’s estimate of gestation based on date of conception.

In addition to sending templates for compiling information on race and ethnicity as separate variables, since 2001, CDC has provided alternative templates for the tabulation of aggregate cross-classified race/ethnicity data. Before 2007, few reporting areas returned these alternative templates; results by these cross-classified race/ethnicity categories (non-Hispanic white, non-Hispanic black, non-Hispanic other, and Hispanic) are thus shown only for 2007–2014.

Finally, both the original and alternative templates provided by CDC request that aggregate numbers for certain variables be cross-tabulated by a second variable. These cross-tabulations include gestational age (separately by maternal age, by method type, by race, by ethnicity, and by race/ethnicity) and maternal age and marital status (separately by race, by ethnicity, and by race/ethnicity).

In this report, medical and surgical abortions are further categorized by gestational age. Early medical abortion in this report is defined as the administration of medication or medications (typically mifepristone followed by misoprostol) to induce an abortion at ≤8 completed weeks’ gestation^¶^; medical abortion at >8 completed weeks’ gestation is defined as the administration of medication or medications (typically serial vaginal prostaglandins sometimes following mifepristone) to induce an abortion at >8 weeks’ gestation. For surgical abortions, abortions are categorized as having been performed at ≤13 weeks’ gestation or at >13 weeks’ gestation because of differences in technique used generally before and after 13 weeks (*26*). Finally, because intrauterine instillations cannot be performed early in gestation, abortions reported to have been performed by intrauterine instillation at ≤12 weeks’ gestation are excluded from calculation of the percentage of abortions by known method type.**

### Measures of Abortion

Four measures of abortion are presented in this report: 1) the total number of abortions in a given population, 2) the percentage of abortions obtained by women in a given population, 3) the abortion rate (number of abortions per 1,000 women aged 15–44 years or other specific group within a given population), and 4) the abortion ratio (number of abortions per 1,000 live births within a given population). Although total numbers and percentages are useful for determining how many women have obtained an abortion, abortion rates adjust for differences in population size and reflect how likely abortion is among women in particular groups. Abortion ratios measure the relative number of pregnancies in a population that end in abortion compared with live birth. Abortion ratios are influenced both by the proportion of pregnancies in a population that are unintended and the proportion of unintended pregnancies that end in abortion. Abortion ratios also are influenced by the proportion of intended pregnancies that end in abortion; however, intended pregnancies account for a very small percentage of abortions (<5%) (*29*).

U.S. Census Bureau estimates of the resident female population of the United States were used as the denominator for calculating abortion rates (*30*–*39*). Overall abortion rates were calculated from the population of women aged 15–44 years living in the reporting areas that provided data. For adolescents aged <15 years, abortion rates were determined on the basis of the number of adolescents aged 13–14 years; similarly, for women aged ≥40 years, abortion rates were determined on the basis of the number of women aged 40–44 years. For the calculation of abortion ratios, live birth data were obtained from CDC natality files (*40*) and included births to women of all ages living in the reporting areas that provided abortion data.

### Data Presentation and Analysis

This report provides state-specific and overall abortion numbers, rates, and ratios for the 49 areas that reported to CDC for 2014 (excludes California, Maryland, and New Hampshire). In addition, this report describes the characteristics of women who obtained abortions in 2014. Because the completeness of reporting on the characteristics of women varies by year and by variable, this report only describes the characteristics of women obtaining abortions in areas that met reporting standards (i.e., reported at least 20 abortions overall, provided data categorized in accordance with surveillance variables, and had <15% unknown values for a given characteristic). Abortion rates and ratios have been omitted for reporting areas with <20 abortions because results are considered unstable (*41*). Cells with a value in the range of 1–4 have been suppressed to maintain confidentiality.

Although most of the data are presented by the reporting area in which the abortions were performed, 48 reporting areas in 2014 also provided the number of abortions by maternal residence.^††^ However, two of these reporting areas (Illinois and Wisconsin) reported certain characteristics for in-state residents but not for out-of-state residents. Four other reporting areas (Iowa, Louisiana, Massachusetts, and New Mexico) provided only the total number of abortions for out-of-state residents without specifying individual states or areas of residence from which these women came. As a result, abortion statistics in this report by area of residence should be interpreted with caution and might underestimate the incidence of abortion, especially for reporting areas from which many women travel to other states to obtain abortion services.

To evaluate overall trends in the number, rate, and ratio of reported abortions, annual data are presented for the 48 areas that reported every year during 2005–2014. Linear regression analysis was used to assess the overall rate of change among these areas during the entire 10-year period of analysis (2005–2014) and during the first and second halves of the period of analysis (2005–2009 and 2010–2014). The percentage change in abortion measures from the most recent past year (2013 to 2014) and from the beginning to the end of the 10-year period of analysis (2005 to 2014) also were calculated for these same 48 areas. Consistent with previous reports, key findings are highlighted to provide observed changes over time and differences between groups. However, comparisons do not infer statistical significance, and lack of comment regarding the difference between values does not imply that no statistically significant difference exists.

For the analysis of certain additional variables (i.e., abortions by maternal age and gestational age), annual data are presented for areas that met reporting standards every year during 2005–2014; the percentage change was calculated from the beginning to the end of the 10-year period of analysis (2005 to 2014), from the beginning to the end of the first and second halves of this period (2005 to 2009 and 2010 to 2014), and from the most recent past year (2013 to 2014). For other variables (i.e., race/ethnicity, method for performing an abortion, marital status, number of previous abortions, and number of previous live births), annual data are not presented; areas were included if they met reporting standards for the years needed for percentage change calculations. To evaluate trends in the use of different methods for performing an abortion, reporting areas were included only if they met reporting standards and if they specifically included medical abortion as a method on their reporting form.

Medical abortions performed at 9 completed weeks are also reported for 2011 to 2014. These data are reported to monitor any changes in clinical practice that might have occurred with the accumulation of evidence on the safety and effectiveness of medical abortion past 63 days of gestation (≤8 completed weeks) (*42*) and changes in professional practice guidelines published in 2013 and 2014 (*43*,*44*). Both of these events preceded the 2016 Food and Drug Administration (FDA) extension of the gestational age limit for the use of mifepristone for early medical abortion to 70 days (≤9 completed weeks) (*45*).

Some of the 49 areas that reported for 2014 are not included in certain trend analyses when data did not meet reporting standards. As a result, summary measures for comparisons over time might differ slightly from the point estimates presented for all areas that reported for 2014.

### Abortion Mortality

CDC has reported data on abortion-related deaths periodically since information on abortion mortality first was included in the 1972 abortion surveillance report (*14*,*46*). An abortion-related death is defined as a death resulting from a direct complication of an abortion (legal or illegal), an indirect complication caused by a chain of events initiated by an abortion, or an aggravation of a pre-existing condition by the physiologic or psychologic effects of abortion (*47*). All deaths determined to be related causally to induced abortion are classified as abortion related regardless of the time between the abortion and death. In addition, any pregnancy-related death in which the pregnancy outcome was induced abortion regardless of the causal relation between the abortion and the death is considered an abortion-related death. An abortion is defined as legal only if it is performed by a licensed clinician within the limits of state law.

Since 1987, CDC has monitored abortion-related deaths through its Pregnancy Mortality Surveillance System (*48*,*49*). Sources of data for abortion-related deaths have included state vital records; media reports, including computerized searches of full-text newspaper and other print media databases; and individual case reports by public health agencies, including maternal mortality review committees, health care providers and provider organizations, private citizens, and citizen groups. For each death that possibly is related to abortion, CDC requests clinical records and autopsy reports. Two medical epidemiologists independently review these reports to determine the cause of death and whether the death was abortion related. Discrepancies are discussed and resolved by consensus. Each death is categorized by abortion type as legal induced, illegal induced, spontaneous, or unknown type.

This report provides data from the Pregnancy Mortality Surveillance System on induced abortion-related deaths that occurred in 2013, the most recent year for which data are available. Data on induced abortion-related deaths that occurred during 1972–2012 already have been published (*14*), and possible abortion-related deaths that occurred during 2014–2017 are being assessed. For 1998–2013, abortion surveillance data reported to CDC cannot be used alone to calculate national case-fatality rates (number of legal induced abortion-related deaths per 100,000 reported legal induced abortions in the United States) because certain states^§§^ did not report abortion data every year during this period. Thus, national legal induced abortion case-fatality rates were calculated with denominator data from a more complete source on the total number of abortions performed in the United States (*50*). Because rates determined on the basis of a numerator of <20 deaths are highly variable (*41*), national legal induced abortion case-fatality rates were calculated for consecutive 5-year periods during 1973–2007 and for a consecutive 6-year period during 2008–2013.

## Results

### U.S. Totals

**FIGURE 1:**
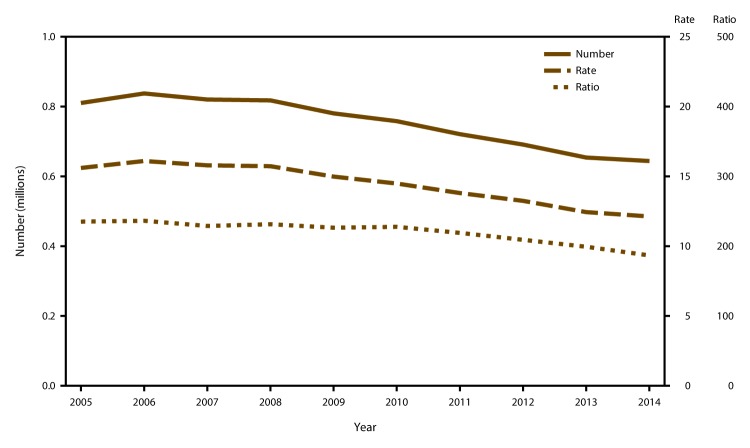
Number, rate,* and ratio^†^ of abortions performed by year — selected reporting areas,^§^ United States, 2005–2014 * Number of abortions per 1,000 women aged 15-44 years. ^†^ Number of abortions per 1,000 live births. ^§^ Data are for 48 reporting areas; excludes California, Louisiana, Maryland, and New Hampshire.

Among the 49 reporting areas that provided data for 2014, a total of 652,639 abortions were reported. Of these abortions, 642,317 (98.4%) were obtained from the 48 reporting areas that provided data every year during 2005–2014.^¶¶^ These same 48 areas had an abortion rate of 12.1 abortions per 1,000 women aged 15–44 years and an abortion ratio of 193 abortions per 1,000 live births (Table 1). From 2013 to 2014, the total number of reported abortions decreased 2% (from 654,458), the abortion rate decreased 2% (from 12.4 abortions per 1,000 women aged 15–44 years), and the abortion ratio decreased 3% (from 199 abortions per 1,000 live births). From 2005 to 2014, among the same 48 areas that reported every year during this period, the total number of reported abortions decreased 21% (from 809,354), the abortion rate decreased 22% (from 15.6 abortions per 1,000 women aged 15–44 years), and the abortion ratio decreased 18% (from 235 abortions per 1,000 live births) (Figure 1). Among these same 48 areas, the annual rate of decrease fitted from the regression analysis was greater during 2010–2014 than during 2005–2009 for all three measures of abortion. During 2010–2014, the number of reported abortions decreased by 29,583 abortions per year, the abortion rate decreased by 0.62 abortions per 1,000 women per year, and the abortion ratio decreased by 8.7 abortions per 1,000 live births per year. In contrast, during 2005–2009, the number of reported abortions decreased by 7,451 abortions per year, the abortion rate decreased by 0.16 abortions per 1,000 women per year, and the abortion ratio decreased by 2.3 abortions per 1,000 live births per year.

**TABLE 1:** Number, percentage, rate,* and ratio^†^ of reported abortions — selected reporting areas, United States, 2005–2014

Year	Selected reporting areas^§^	Continuously reporting areas^¶^		
	No.	No. (%)**	Rate	Ratio
2005	820,151	809,354 (98.7)	15.6	235
2006	852,385^††^	836,651 (98.2)	16.1	236
2007	827,609	820,776 (99.2)	15.8	229
2008	825,564	818,748 (99.2)	15.7	231
2009	789,217^§§^	781,050 (99.0)	15.0	226
2010	765,651	756,779 (98.8)	14.5	227
2011	730,322	721,367 (98.8)	13.8	218
2012	699,202	689,977 (98.7)	13.2	209
2013	664,435	654,458 (98.5)	12.4	199
2014	652,639	642,317 (98.4)	12.1	193

* Number of abortions per 1,000 women aged 15–44 years.

^†^ Number of abortions per 1,000 live births.

^§^ For each given year, excludes reporting areas that did not report that year’s abortion numbers to CDC: California (2005–2014), Louisiana (2005), Maryland (2007–2014), and New Hampshire (2005–2014).

^¶^ For all years, excludes reporting areas that did not report abortion numbers every year during the period of analysis (2005-2014): California, Louisiana, Maryland, and New Hampshire.

** Abortions from areas that reported every year during 2005–2014 as a percentage of all reported abortions.

^††^ This number is greater than reported in the 2006 report because of numbers subsequently provided by Louisiana.

^§§^ This number is greater than reported in the 2009 report because of numbers subsequently provided by Delaware.

### Occurrence and Residence

Abortion numbers, rates, and ratios for 2014 have been calculated by reporting area of occurrence and the residence of the women who obtained the abortions (Table 2). By reporting area of occurrence, a considerable range existed in the abortion rate (from 3.5 abortions per 1,000 women aged 15–44 years in South Dakota to 23.9 abortions per 1,000 women in New York [city and state combined]) and the abortion ratio (from 45 abortions per 1,000 live births in South Dakota to 405 abortions per 1,000 live births in New York [city and state combined]).*** Similarly, a considerable range existed by residence^†††^ in the abortion rate (from 4.5 abortions per 1,000 women aged 15–44 years in Utah to 23.2 abortions per 1,000 women aged 15–44 years in New York [city and state combined]) and the abortion ratio (from 57 abortions per 1,000 live births in Utah to 394 abortions per 1,000 live births in New York [city and state combined]). Because of variation that occurred among reporting areas in the percentage of abortions obtained by out-of-state residents (from 0.9% in Hawaii to 55.6% in the District of Columbia), abortion rates and ratios calculated by maternal residence might provide a more accurate reflection of the state-specific distribution of women obtaining abortions. However, because states vary in the level of detail they collect on maternal residence, 12.2% of abortions were reported to CDC without exact information on maternal residence.

**TABLE 2:** Number, rate,* and ratio^†^ of reported abortions, by reporting area of residence and occurrence and by percentage of abortions obtained by out-of-state residents — United States, 2014

State/Area	Residence			Occurrence			% obtained by out-of-state residents^§^
	No.	Rate	Ratio	No.	Rate	Ratio	
Alabama	7,893	8.2	133	8,080	8.4	136	17.7
Alaska	1,647	11.2	145	1,518	10.3	133	1.0
Arizona	12,914	9.9	149	12,900	9.9	148	1.2
Arkansas	4,024	7.0	104	4,253	7.4	110	22.2
California^¶^	—	—	—	—	—	—	—
Colorado	9,453	8.7	144	10,648	9.8	162	11.3
Connecticut	10,625	15.5	293	10,611	15.5	292	2.6
Delaware	2,920	16.2	266	2,937	16.3	268	16.0
District of Columbia**	1,407	7.9	148	2,790	15.7	293	55.6
Florida^††^	—	—	—	72,107	19.6	328	—
Georgia	26,563	12.6	203	30,013	14.3	229	12.3
Hawaii	2,011	7.5	108	2,147	8.0	116	0.9
Idaho	1,767	5.6	77	1,353	4.3	59	5.2
Illinois	33,918	13.1	214	38,472	14.8	243	8.2
Indiana	9,765	7.5	116	8,118	6.3	97	6.1
Iowa^§§^	3,766	6.4	95	4,020	6.9	101	13.8
Kansas	3,779	6.7	96	7,219	12.9	184	49.6
Kentucky	4,923	5.8	88	3,442	4.0	61	11.3
Louisiana^§§^	9,416	10.0	146	10,322	10.9	160	14.1
Maine	1,939	8.3	153	2,021	8.6	159	3.6
Maryland^¶^	—	—	—	—	—	—	—
Massachusetts^§§^	18,630	13.6	259	19,354	14.1	269	3.7
Michigan	26,646	14.1	233	27,629	14.6	242	4.7
Minnesota	9,533	9.1	136	10,123	9.6	145	9.3
Mississippi	5,104	8.5	132	2,303	3.8	59	3.6
Missouri	8,935	7.6	119	5,060	4.3	67	8.8
Montana	1,504	8.1	121	1,690	9.1	136	13.4
Nebraska	2,098	5.8	78	2,270	6.2	85	12.1
Nevada	7,870	13.9	219	8,132	14.4	227	3.9
New Hampshire^¶^	—	—	—	—	—	—	—
New Jersey^¶¶^	24,454	14.2	237	24,181	14.0	234	5.2
New Mexico^§§^	3,655	9.2	140	4,500	11.3	173	21.0
New York	93,984	23.2	394	96,711	23.9	405	3.3
New York City	NA	NA	NA	67,620	34.8	575	7.9
New York State	NA	NA	NA	29,091	13.8	240	5.2
North Carolina	21,385	10.8	177	24,605	12.4	203	14.5
North Dakota	1,009	7.0	89	1,264	8.8	111	30.3
Ohio	21,650	9.8	155	21,186	9.6	152	5.5
Oklahoma	4,808	6.3	90	4,916	6.4	92	8.7
Oregon	7,683	9.9	169	8,231	10.6	181	9.6
Pennsylvania	32,683	13.6	230	32,126	13.3	226	4.2
Rhode Island	2,581	12.3	238	2,990	14.2	276	16.0
South Carolina	9,774	10.4	170	5,714	6.1	99	5.3
South Dakota	755	4.8	61	551	3.5	45	13.6
Tennessee	10,987	8.5	135	12,373	9.5	152	21.3
Texas	54,401	9.6	136	54,148	9.6	135	1.9
Utah	2,905	4.5	57	2,948	4.6	58	6.1
Vermont	1,161	10.0	189	1,235	10.6	201	6.8
Virginia	20,444	12.1	198	20,187	12.0	195	5.9
Washington	17,583	12.6	198	17,710	12.7	200	4.9
West Virginia	1,884	5.6	93	1,730	5.1	85	13.2
Wisconsin	7,014	6.5	104	5,800	5.3	86	2.8
Wyoming	642	5.8	83	—***	—^†††^	—^†††^	—^†††^
Canada	100	NA	NA	NA	NA	NA	NA
Mexico	191	NA	NA	NA	NA	NA	NA
Other country or territory	46	NA	NA	NA	NA	NA	NA
**Total known**	**573,199**	**NA**	**NA**	**NA**	**NA**	**NA**	**NA**
**Percentage reported by known residence**	**87.8**	**NA**	**NA**	**NA**	**NA**	**NA**	**NA**
**Total unknown residence**	**79,440**	**NA**	**NA**	**NA**	**NA**	**NA**	**NA**
Out of state, exact residence not stated	3,438	NA	NA	NA	NA	NA	NA
No information on residence provided	76,002	NA	NA	NA	NA	NA	NA
**Percentage reported by unknown residence**	**12.2**	**NA**	**NA**	**NA**	**NA**	**NA**	**NA**
**Total**	**652,639**	**NA**	**NA**	**NA**	**NA**	**NA**	**NA**

**Abbreviation:** NA = not applicable.

* Number of abortions per 1,000 women aged 15–44 years.

^†^ Number of abortions per 1,000 live births.

^§^ Additional details on the state in which abortions were provided, cross-tabulated by the state of maternal residence, are available at http://www.cdc.gov/reproductivehealth/data_stats/Abortion.htm.

^¶^ Reporting area did not report; because numbers for this area are available only from other reporting areas where residents obtained abortions, meaningful statistics cannot be reported.

** Because reporting is not mandatory, a complete number of abortions performed in the District of Columbia could not be obtained.

^††^ Reported by occurrence only; because abortion numbers by residence for Florida are available only from other states where residents obtained abortions, meaningful statistics cannot be reported.

^§§^ Reporting area reported abortion numbers for both in-state and out-of-state residents; for out-of-state residents, the state or area of residence was not provided.

^¶¶^ Data from hospitals and licensed ambulatory care facilities only; because reporting is not mandatory for private physicians and women’s centers, a complete number of abortions performed in New Jersey could not be obtained.

*** Total abortion number <20.

^†††^ Abortion rates and ratios and percentage of abortions obtained by out-of-state residents were not calculated for Wyoming because results based on a small number of abortions are unstable.

### Maternal Age

**FIGURE 2:**
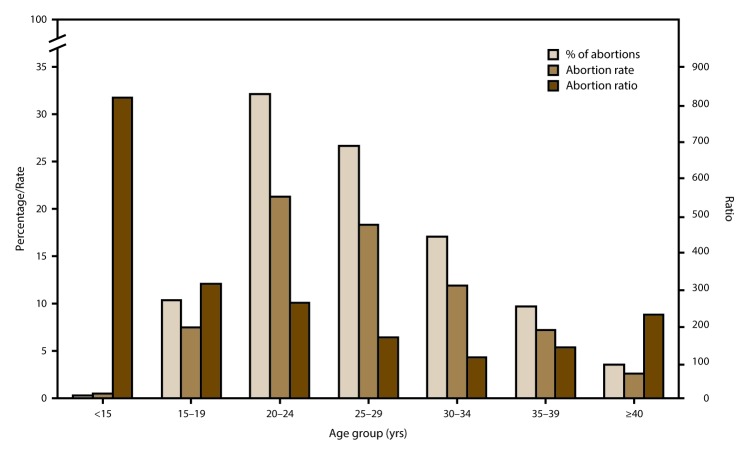
Percentage of total abortions, abortion rate,* and abortion ratio,^†^ by age group of women who obtained a legal abortion — selected reporting areas,^§^ United States, 2014 * Number of abortions per 1,000 women aged 15-44 years. ^†^ Number of abortions per 1,000 live births. ^§^ Data are for 46 areas; excludes six areas (California, Florida, Maryland, New Hampshire, Texas, and Wyoming) that did not report, did not report by age, or did not meet reporting standards.

Among the 46 areas that reported by maternal age for 2014, women in their 20s accounted for the majority (58.9%) of abortions and had the highest abortion rates (21.3 and 18.4 abortions per 1,000 women aged 20–24 and 25–29 years, respectively) (Figure 2) (Table 3). Women in the youngest (<15 years) and oldest (≥40 years) age groups accounted for the smallest percentages of abortions (0.3% and 3.6%, respectively) and had the lowest abortion rates (0.5 and 2.6 abortions per 1,000 women aged <15 and ≥40 years, respectively). Among the 42 reporting areas that provided data by maternal age every year during 2005–2014, this pattern across age groups was stable, with the majority of abortions and the highest abortion rates occurring among women aged 20–29 years and the lowest percentages of abortions and abortion rates occurring among women in the youngest and oldest age groups (Table 4). From 2005 to 2014, abortion rates decreased among all age groups <40 years, although the decreases for adolescents (62% and 49% for adolescents aged <15 and 15–19 years, respectively) were greater than the decreases for women aged 20–39 years (5%–27%). For all age groups, including women aged ≥40 years, decreases in the abortion rate were greater from 2010 to 2014 than from 2005 to 2009, and these decreases continued from 2013 to 2014 for all age groups <25 years.

**TABLE 3:** Reported abortions, by known age group and reporting area of occurrence — selected reporting areas,* United States, 2014

State/Area	Age group (yrs)							Total abortions reported by known age
	<15	15–19	20–24	25–29	30–34	35–39	≥40	
	No. (%)^†^	No. (%)	No. (%)	No. (%)	No. (%)	No. (%)	No. (%)	No. (% of all reported abortions)^§^
Alabama	34 (0.4)	954 (11.8)	2,809 (34.8)	2,138 (26.5)	1,298 (16.1)	638 (7.9)	206 (2.6)	**8,077 (100.0)**
Alaska	6 (0.4)	199 (13.1)	494 (32.5)	406 (26.7)	242 (15.9)	115 (7.6)	56 (3.7)	**1,518 (100.0)**
Arizona	22 (0.2)	1,247 (9.7)	4,218 (32.7)	3,389 (26.3)	2,165 (16.8)	1,308 (10.1)	551 (4.3)	**12,900 (100.0)**
Arkansas	13 (0.3)	473 (11.1)	1,364 (32.1)	1,148 (27.0)	724 (17.0)	371 (8.7)	159 (3.7)	**4,252 (100.0)**
Colorado	31 (0.3)	1,124 (10.6)	3,474 (32.7)	2,845 (26.8)	1,784 (16.8)	934 (8.8)	422 (4.0)	**10,614 (99.7)**
Connecticut	27 (0.3)	1,125 (10.9)	3,335 (32.2)	2,765 (26.7)	1,776 (17.2)	961 (9.3)	356 (3.4)	**10,345 (97.5)**
Delaware	9 (0.3)	366 (12.5)	992 (33.8)	722 (24.6)	488 (16.6)	261 (8.9)	99 (3.4)	**2,937 (100.0)**
District of Columbia^¶^	7 (0.3)	312 (11.2)	980 (35.1)	732 (26.2)	430 (15.4)	230 (8.2)	99 (3.5)	**2,790 (100.0)**
Georgia	123 (0.4)	2,945 (9.8)	9,465 (31.5)	8,001 (26.7)	5,403 (18.0)	2,973 (9.9)	1,103 (3.7)	**30,013 (100.0)**
Hawaii	—**	248 (11.6)	699 (32.7)	537 (25.1)	353 (16.5)	220 (10.3)	—	**2,139 (99.6)**
Idaho	7 (0.5)	175 (12.9)	462 (34.1)	339 (25.1)	201 (14.9)	124 (9.2)	45 (3.3)	**1,353 (100.0)**
Illinois^††^	109 (0.3)	3,664 (11.1)	10,654 (32.1)	8,627 (26.0)	5,639 (17.0)	3,228 (9.7)	1,234 (3.7)	**33,155 (99.7)**
Indiana	21 (0.3)	907 (11.2)	2,698 (33.2)	2,164 (26.7)	1,311 (16.1)	767 (9.4)	250 (3.1)	**8,118 (100.0)**
Iowa	13 (0.3)	478 (11.9)	1,334 (33.2)	985 (24.5)	681 (16.9)	377 (9.4)	150 (3.7)	**4,018 (100.0)**
Kansas	16 (0.2)	760 (10.5)	2,341 (32.4)	1,896 (26.3)	1,240 (17.2)	717 (9.9)	249 (3.4)	**7,219 (100.0)**
Kentucky	17 (0.5)	408 (11.9)	1,098 (31.9)	874 (25.4)	575 (16.7)	349 (10.1)	121 (3.5)	**3,442 (100.0)**
Louisiana	45 (0.4)	960 (9.3)	3,307 (32.0)	2,996 (29.0)	1,838 (17.8)	888 (8.6)	288 (2.8)	**10,322 (100.0)**
Maine	—	233 (11.5)	660 (32.7)	539 (26.7)	326 (16.2)	172 (8.5)	—	**2,018 (99.9)**
Massachusetts	37 (0.2)	1,780 (9.2)	6,115 (31.6)	5,255 (27.2)	3,383 (17.5)	1,985 (10.3)	798 (4.1)	**19,353 (100.0)**
Michigan	60 (0.2)	2,967 (10.8)	9,780 (35.5)	7,048 (25.6)	4,353 (15.8)	2,470 (9.0)	865 (3.1)	**27,543 (99.7)**
Minnesota	32 (0.3)	942 (9.3)	3,136 (31.0)	2,756 (27.2)	1,821 (18.0)	1,037 (10.2)	399 (3.9)	**10,123 (100.0)**
Mississippi	11 (0.5)	259 (11.3)	821 (35.7)	623 (27.1)	373 (16.2)	168 (7.3)	47 (2.0)	**2,302 (100.0)**
Missouri	14 (0.3)	555 (11.0)	1,791 (35.4)	1,275 (25.2)	789 (15.6)	472 (9.3)	161 (3.2)	**5,057 (99.9)**
Montana	—	229 (13.6)	573 (33.9)	437 (25.9)	259 (15.3)	137 (8.1)	—	**1,689 (99.9)**
Nebraska	10 (0.4)	221 (9.7)	748 (33.0)	593 (26.1)	387 (17.0)	221 (9.7)	90 (4.0)	**2,270 (100.0)**
Nevada	10 (0.1)	753 (9.4)	2,364 (29.5)	2,288 (28.5)	1,578 (19.7)	729 (9.1)	293 (3.7)	**8,015 (98.6)**
New Jersey^§§^	53 (0.2)	2,220 (9.2)	7,114 (29.5)	6,812 (28.2)	4,235 (17.6)	2,594 (10.8)	1,102 (4.6)	**24,130 (99.8)**
New Mexico	26 (0.6)	596 (14.0)	1,369 (32.2)	1,100 (25.9)	686 (16.1)	352 (8.3)	126 (3.0)	**4,255 (94.6)**
New York	289 (0.3)	10,390 (10.8)	29,452 (30.5)	25,811 (26.7)	17,158 (17.8)	9,816 (10.2)	3,701 (3.8)	**96,617 (99.9)**
New York City	199 (0.3)	6,868 (10.2)	19,764 (29.2)	18,345 (27.1)	12,462 (18.4)	7,262 (10.7)	2,718 (4.0)	**67,618 (100.0)**
New York State	90 (0.3)	3,522 (12.1)	9,688 (33.4)	7,466 (25.7)	4,696 (16.2)	2,554 (8.8)	983 (3.4)	**28,999 (99.7)**
North Carolina	75 (0.3)	2,266 (9.8)	7,717 (33.4)	6,201 (26.9)	3,895 (16.9)	2,129 (9.2)	794 (3.4)	**23,077 (93.8)**
North Dakota	—	128 (10.1)	447 (35.4)	345 (27.3)	188 (14.9)	113 (8.9)	—	**1,264 (100.0)**
Ohio	77 (0.4)	2,253 (10.6)	7,157 (33.8)	5,590 (26.4)	3,459 (16.3)	1,967 (9.3)	659 (3.1)	**21,162 (99.9)**
Oklahoma	25 (0.5)	630 (12.8)	1,571 (32.0)	1,266 (25.8)	845 (17.2)	397 (8.1)	173 (3.5)	**4,907 (99.8)**
Oregon	22 (0.3)	864 (10.8)	2,445 (30.5)	2,128 (26.6)	1,396 (17.4)	818 (10.2)	334 (4.2)	**8,007 (97.3)**
Pennsylvania	110 (0.3)	3,332 (10.4)	10,848 (33.8)	8,750 (27.2)	5,162 (16.1)	2,911 (9.1)	1,013 (3.2)	**32,126 (100.0)**
Rhode Island	7 (0.2)	252 (8.5)	1,020 (34.2)	775 (26.0)	513 (17.2)	295 (9.9)	117 (3.9)	**2,979 (99.6)**
South Carolina	13 (0.2)	639 (11.2)	1,817 (31.8)	1,510 (26.4)	1,008 (17.6)	543 (9.5)	184 (3.2)	**5,714 (100.0)**
South Dakota	—	62 (11.3)	167 (30.3)	150 (27.2)	87 (15.8)	66 (12.0)	—	**551 (100.0)**
Tennessee	56 (0.5)	1,210 (10.3)	3,883 (33.0)	3,247 (27.6)	1,938 (16.5)	1,102 (9.4)	315 (2.7)	**11,751 (95.0)**
Utah	9 (0.3)	347 (11.8)	930 (31.7)	695 (23.7)	502 (17.1)	338 (11.5)	111 (3.8)	**2,932 (99.5)**
Vermont	—	153 (12.4)	406 (33.0)	311 (25.3)	189 (15.4)	117 (9.5)	—	**1,231 (99.7)**
Virginia	44 (0.2)	1,627 (8.1)	6,340 (31.5)	5,516 (27.4)	3,667 (18.2)	2,121 (10.5)	795 (4.0)	**20,110 (99.6)**
Washington	37 (0.2)	1,966 (11.1)	5,579 (31.5)	4,601 (26.0)	3,072 (17.4)	1,775 (10.0)	669 (3.8)	**17,699 (99.9)**
West Virginia	9 (0.5)	210 (12.1)	564 (32.6)	450 (26.0)	274 (15.8)	186 (10.8)	37 (2.1)	**1,730 (100.0)**
Wisconsin^††^	15 (0.3)	642 (11.4)	1,892 (33.5)	1,473 (26.1)	902 (16.0)	515 (9.1)	201 (3.6)	**5,640 (100.0)**
**Total**	**1,557 (0.3)**	**54,071 (10.4)**	**166,430 (32.2)**	**138,109 (26.7)**	**88,593 (17.1)**	**50,007 (9.7)**	**18,697 (3.6)**	**517,464 (99.3)^¶¶^**
Abortion rate***	**0.5**	**7.5**	**21.3**	**18.4**	**11.9**	**7.2**	**2.6**	**11.7**
**Abortion ratio** ^†††^	**838**	**321**	**270**	**172**	**119**	**148**	**245**	**188**

* Data from 46 reporting areas; excludes six reporting areas (California, Florida, Maryland, New Hampshire, Texas, and Wyoming) that did not report, did not report by age, or did not meet reporting standards.

^†^ Percentages for the individual component categories might not add to 100 because of rounding.

^§^ Percentage is calculated as the number of abortions reported by known age divided by the sum of abortions reported by known and unknown age.

^¶^ Because reporting is not mandatory, a complete number of abortions performed in the District of Columbia could not be obtained.

** Cell details are not displayed because of small numbers (n = 1–4).

^††^ Includes residents only.

^§§^ Data from hospitals and licensed ambulatory care facilities only; because reporting is not mandatory for private physicians and women’s centers, information could not be obtained for all abortions performed in New Jersey.

^¶¶^ Percentage is based on 520,998 abortions reported among the areas that met reporting standards for age.

*** Number of abortions obtained by women in a given age group per 1,000 women in that same age group. Women aged 13–14 years were used as the denominator for the group of women aged <15 years, and women aged 40–44 years were used as the denominator for the group of women aged ≥40 years. Women aged 15–44 years were used as the denominator for the overall rate. For each reporting area, abortions for women of unknown age were distributed according to the distribution of abortions among women of known age for that area.

^†††^ Number of abortions obtained by women in a given age group per 1,000 live births to women in that same age group. For each reporting area, abortions for women of unknown age were distributed according to the distribution of abortions among women of known age for that area.

**TABLE 4:** Reported abortions, by known age group and year — selected reporting areas,* United States, 2005–2014

Age group (yrs)	Year										% change			
	2005	2006	2007	2008	2009	2010	2011	2012	2013	2014	2005 to 2009	2010 to 2014	2013 to 2014	2005 to 2014
**% of abortions**														
<15	0.6	0.6	0.6	0.5	0.5	0.5	0.4	0.4	0.3	0.3	-16.7	-40.0	0.0	-50.0
15–19	17.0	17.0	17.0	16.6	16.0	15.0	13.8	12.6	11.5	10.5	-5.9	-30.0	-8.7	-38.2
20–24	32.7	32.5	32.5	32.6	32.5	32.9	33.0	32.9	32.8	32.1	-0.6	-2.4	-2.1	-1.8
25–29	23.3	23.9	24.0	24.2	24.2	24.2	24.6	25.1	25.8	26.6	3.9	9.9	3.1	14.2
30–34	14.5	14.1	14.0	14.3	14.7	15.2	15.7	16.3	16.8	17.1	1.4	12.5	1.8	17.9
35–39	8.8	8.9	8.8	8.8	8.8	8.8	8.9	9.1	9.2	9.7	0.0	10.2	5.4	10.2
≥40	3.2	3.1	3.1	3.1	3.3	3.4	3.6	3.7	3.6	3.6	3.1	5.9	0.0	12.5
**Abortion rate^†^**														
<15	1.3	1.3	1.3	1.2	1.1	1.0	0.9	0.8	0.6	0.5	-15.4	-50.0	-16.7	-61.5
15–19	14.8	15.0	14.6	14.3	13.1	12.0	10.8	9.5	8.3	7.5	-11.5	-37.5	-9.6	-49.3
20–24	29.1	29.7	29.2	29.3	27.7	26.7	25.1	23.5	22.0	21.3	-4.8	-20.2	-3.2	-26.8
25–29	22.0	22.5	21.8	21.8	20.7	20.1	19.4	18.8	18.3	18.5	-5.9	-8.0	1.1	-15.9
30–34	13.5	13.8	13.7	13.9	13.4	13.2	12.7	12.4	11.9	11.9	-0.7	-9.8	0.0	-11.9
35–39	7.7	7.9	7.8	7.9	7.7	7.6	7.5	7.3	7.0	7.3	0.0	-3.9	4.3	-5.2
≥40	2.5	2.6	2.6	2.6	2.7	2.7	2.8	2.7	2.6	2.6	8.0	-3.7	0.0	4.0
**Abortion ratio** ^§^														
<15	884	878	908	941	994	1004	972	940	915	859	12.4	-14.4	-6.1	-2.8
15–19	393	385	370	372	362	367	360	338	327	324	-7.9	-11.7	-0.9	-17.6
20–24	292	288	283	293	292	300	294	283	274	272	0.0	-9.3	-0.7	-6.8
25–29	188	190	184	188	185	184	179	175	171	172	-1.6	-6.5	0.6	-8.5
30–34	141	141	137	140	138	136	131	127	122	118	-2.1	-13.2	-3.3	-16.3
35–39	170	172	169	173	172	170	163	157	147	148	1.2	-12.9	0.7	-12.9
≥40	279	278	274	267	271	267	268	265	247	245	-2.9	-8.2	-0.8	-12.2
**Total (no.)**	**626,550**	**644,561**	**635,106**	**638,653**	**610,146**	**589,393**	**562,328**	**537,137**	**509,107**	**504,623**	—	—	—	—

* Data from 42 reporting areas; by year, these reporting areas represent 86%–88% of all abortions reported to CDC by age during 2005–2014. Excludes 10 reporting areas (California, District of Columbia, Florida, Louisiana, Maine, Maryland, New Hampshire, Texas, Vermont, and Wyoming) that did not report, did not report by age, or did not meet reporting standards for ≥1 year.

^†^ Number of abortions obtained by women in a given age group per 1,000 women in that same age group. Women aged 13–14 years were used as the denominator for the group of women aged <15 years, and women aged 40–44 years were used as the denominator for the group of women aged ≥40 years. Women aged 15–44 years were used as the denominator for the overall rate. For each reporting area, abortions for women of unknown age were distributed according to the distribution of abortions among women of known age for that area.

^§^ Number of abortions obtained by women in a given age group per 1,000 live births to women in that same age group. For each reporting area, abortions for women of unknown age were distributed according to the distribution of abortions among women of known age for that area.

In contrast to the percentage of abortions and abortion rates, abortion ratios in 2014 were highest among adolescents aged ≤19 years and lowest among women aged 30–39 years (Figure 2) (Table 3). Among the 42 reporting areas that provided data by maternal age for every year during 2005–2014, abortion ratios decreased among women in all age groups. The abortion ratio increased 12% for adolescents aged <15 years from 2005 to 2009 but decreased for all age groups from 2010 to 2014. In addition, for every age group with declines for both periods, the declines that occurred from 2010 to 2014 exceeded the declines from 2005 to 2009 (Table 4).

### Adolescents

Among the 43 areas that reported maternal age by individual year among adolescents for 2014, adolescents aged 18–19 years accounted for the majority (66.8%) of adolescent abortions and had the highest adolescent abortion rates (10.6 and 14.3 abortions per 1,000 adolescents aged 18 and 19 years, respectively); adolescents aged <15 years accounted for the smallest percentage of adolescent abortions (2.8%) and had the lowest adolescent abortion rate (0.5 abortions per 1,000 adolescents aged 13–14 years) (Table 5). Among the 38 reporting areas that provided maternal age data for adolescents for each individual year of reporting during 2005–2014, the percentage of abortions accounted for by adolescents aged 18 and 19 years increased, whereas the percentage of abortions accounted for by adolescents aged <18 years decreased (Table 6). Among adolescents aged <18 years, abortion rates decreased by 57%–62%; among adolescents aged 18 and 19 years, the abortion rates decreased 50% and 43%, respectively. For all age groups, decreases in the abortion rate were greater from 2010 to 2014 than from 2005 to 2009, and these decreases continued from 2013 to 2014.

**TABLE 5:** Reported abortions among adolescents, by known age and reporting area of occurrence — selected reporting areas,* United States, 2014

State/Area	Age (yrs)						Total no.
	<15	15	16	17	18	19	
	No. (%)^†^	No. (%)	No. (%)	No. (%)	No. (%)	No. (%)	
Alabama	34 (3.4)	49 (5.0)	108 (10.9)	138 (14.0)	268 (27.1)	391 (39.6)	**988**
Alaska	6 (2.9)	10 (4.9)	17 (8.3)	27 (13.2)	67 (32.7)	78 (38.0)	**205**
Arizona	22 (1.7)	61 (4.8)	112 (8.8)	162 (12.8)	412 (32.5)	500 (39.4)	**1,269**
Arkansas	13 (2.7)	39 (8.0)	47 (9.7)	68 (14.0)	143 (29.4)	176 (36.2)	**486**
Colorado	31 (2.7)	63 (5.5)	107 (9.3)	169 (14.6)	344 (29.8)	441 (38.2)	**1,155**
Connecticut	27 (2.3)	57 (4.9)	127 (11.0)	201 (17.4)	307 (26.6)	433 (37.6)	**1,152**
Delaware	9 (2.4)	17 (4.5)	36 (9.6)	68 (18.1)	114 (30.4)	131 (34.9)	**375**
District of Columbia^¶^	7 (2.2)	14 (4.4)	32 (10.0)	80 (25.1)	75 (23.5)	111 (34.8)	**319**
Georgia	123 (4.0)	193 (6.3)	291 (9.5)	444 (14.5)	821 (26.8)	1,196 (39.0)	**3,068**
Hawaii	—^§^	—	25 (10.0)	48 (19.3)	66 (26.5)	98 (39.4)	**249**
Idaho	—	—	14 (7.7)	21 (11.5)	60 (33.0)	76 (41.8)	**182**
Indiana	21 (2.3)	40 (4.3)	92 (9.9)	137 (14.8)	270 (29.1)	368 (39.7)	**928**
Iowa	13 (2.6)	25 (5.1)	51 (10.4)	91 (18.5)	119 (24.2)	192 (39.1)	**491**
Kansas	16 (2.1)	39 (5.0)	59 (7.6)	99 (12.8)	239 (30.8)	324 (41.8)	**776**
Kentucky	17 (4.0)	26 (6.1)	41 (9.6)	75 (17.6)	117 (27.5)	149 (35.1)	**425**
Louisiana	45 (4.5)	60 (6.0)	93 (9.3)	164 (16.3)	249 (24.8)	394 (39.2)	**1,005**
Maine	—	—	24 (10.1)	51 (21.5)	75 (31.6)	78 (32.9)	**237**
Michigan	60 (2.0)	158 (5.2)	270 (8.9)	429 (14.2)	860 (28.4)	1,250 (41.3)	**3,027**
Minnesota	32 (3.3)	54 (5.5)	81 (8.3)	140 (14.4)	243 (24.9)	424 (43.5)	**974**
Mississippi	11 (4.1)	15 (5.6)	25 (9.3)	36 (13.3)	75 (27.8)	108 (40.0)	**270**
Missouri	14 (2.5)	32 (5.6)	47 (8.3)	69 (12.1)	169 (29.7)	238 (41.8)	**569**
Montana	—	—	19 (8.2)	40 (17.2)	79 (33.9)	79 (33.9)	**233**
Nebraska	10 (4.3)	11 (4.8)	25 (10.8)	26 (11.3)	65 (28.1)	94 (40.7)	**231**
Nevada	10 (1.3)	31 (4.1)	68 (8.9)	120 (15.7)	225 (29.5)	309 (40.5)	**763**
New Jersey**	53 (2.3)	118 (5.2)	232 (10.2)	409 (18.0)	598 (26.3)	863 (38.0)	**2,273**
New Mexico	26 (4.2)	36 (5.8)	81 (13.0)	113 (18.2)	158 (25.4)	208 (33.4)	**622**
New York	289 (2.7)	533 (5.0)	1,032 (9.7)	1,867 (17.5)	2,944 (27.6)	4,014 (37.6)	**10,679**
New York City	199 (2.8)	352 (5.0)	710 (10.0)	1,251 (17.7)	1,920 (27.2)	2,635 (37.3)	**7,067**
New York State	90 (2.5)	181 (5.0)	322 (8.9)	616 (17.1)	1,024 (28.3)	1,379 (38.2)	**3,612**
North Carolina	75 (3.2)	135 (5.8)	227 (9.7)	303 (12.9)	681 (29.1)	920 (39.3)	**2,341**
North Dakota	—	—	13 (9.9)	14 (10.7)	41 (31.3)	54 (41.2)	**131**
Ohio	77 (3.3)	143 (6.1)	250 (10.7)	360 (15.5)	627 (26.9)	873 (37.5)	**2,330**
Oklahoma	25 (3.8)	38 (5.8)	74 (11.3)	92 (14.0)	181 (27.6)	245 (37.4)	**655**
Oregon	22 (2.5)	56 (6.3)	101 (11.4)	147 (16.6)	260 (29.3)	300 (33.9)	**886**
Rhode Island	7 (2.7)	6 (2.3)	23 (8.9)	38 (14.7)	71 (27.4)	114 (44.0)	**259**
South Carolina	13 (2.0)	24 (3.7)	61 (9.4)	133 (20.4)	174 (26.7)	247 (37.9)	**652**
South Dakota	—	—	8 (12.3)	13 (20.0)	16 (24.6)	21 (32.3)	**65**
Tennessee	56 (4.4)	56 (4.4)	104 (8.2)	145 (11.5)	373 (29.5)	532 (42.0)	**1,266**
Utah	9 (2.5)	17 (4.8)	31 (8.7)	40 (11.2)	98 (27.5)	161 (45.2)	**356**
Vermont	—	—	10 (6.5)	31 (20.1)	44 (28.6)	64 (41.6)	**154**
Virginia	44 (2.6)	81 (4.8)	146 (8.7)	197 (11.8)	464 (27.8)	739 (44.2)	**1,671**
Washington	37 (1.8)	97 (4.8)	195 (9.7)	346 (17.3)	606 (30.3)	722 (36.0)	**2,003**
West Virginia	9 (4.1)	7 (3.2)	19 (8.7)	30 (13.7)	67 (30.6)	87 (39.7)	**219**
Wisconsin^††^	15 (2.3)	34 (5.2)	58 (8.8)	89 (13.5)	196 (29.8)	265 (40.3)	**657**
**Total**	**1,301 (2.8)**	**2,421 (5.2)**	**4,476 (9.6)**	**7,270 (15.6)**	**13,061 (28.0)**	**18,067 (38.8)**	**46,596**
**Abortion rate** ^§§^	**0.5**	**2.0**	**3.7**	**6.0**	**10.6**	**14.3**	
**Abortion ratio** ^¶¶^	**787**	**522**	**391**	**320**	**317**	**263**	

* Data from 43 reporting areas; excludes nine reporting areas (California, Florida, Illinois, Maryland, Massachusetts, New Hampshire, Pennsylvania, Texas, and Wyoming) that did not report, did not report age among adolescents by individual year, or did not meet reporting standards.

^†^ Percentages for the individual component categories might not add to 100 because of rounding.

^§^ Cell details are not displayed because of small numbers (n = 1–4).

^¶^ Because reporting is not mandatory, a complete number of abortions performed in the District of Columbia could not be obtained.

** Data from hospitals and licensed ambulatory care facilities only; because reporting is not mandatory for private physicians and women’s centers, information could not be obtained for all abortions performed in New Jersey.

^††^ Includes residents only.

^§§^ Number of abortions obtained by adolescents in a given age group per 1,000 adolescents in that same age group. Adolescents aged 13–14 years were used as the denominator for adolescents aged <15 years.

^¶¶^ Number of abortions obtained by adolescents in a given age group per 1,000 live births to adolescents in that same age group.

**TABLE 6:** Reported abortions among adolescents, by known age and year — selected reporting areas,* United States, 2005–2014

Age (yrs)	Year										% change			
	2005	2006	2007	2008	2009	2010	2011	2012	2013	2014	2005 to 2009	2010 to 2014	2013 to 2014	2005 to 2014
**% of abortions**														
<15	3.5	3.2	3.3	3.1	3.1	3.2	3.1	3.2	2.9	2.8	-11.4	-12.5	-3.4	**-20.0**
15	6.6	6.2	6.0	5.9	5.7	5.9	5.6	5.6	5.2	5.2	-13.6	-11.9	0.0	**-21.2**
16	11.7	11.9	11.6	11.0	10.8	10.6	10.2	10.0	9.6	9.6	-7.7	-9.4	0.0	**-17.9**
17	17.5	17.6	17.8	17.6	17.2	16.7	16.3	16.0	15.5	15.5	-1.7	-7.2	0.0	**-11.4**
18	27.7	28.0	28.1	28.3	28.1	27.6	28.2	27.9	27.8	28.1	1.4	1.8	1.1	**1.4**
19	33.0	33.0	33.2	34.0	35.1	35.9	36.5	37.2	38.9	38.8	6.4	8.1	-0.3	**17.6**
**Abortion rate^†^**														
<15	1.3	1.3	1.3	1.2	1.1	1.0	0.9	0.8	0.6	0.5	-15.4	-50.0	-16.7	**-61.5**
15	4.9	4.7	4.5	4.4	4.0	3.8	3.2	2.7	2.2	2.0	-18.4	-47.4	-9.1	**-59.2**
16	8.9	8.9	8.6	8.0	7.3	6.6	5.7	4.9	4.0	3.7	-18.0	-43.9	-7.5	**-58.4**
17	13.6	13.7	13.0	12.7	11.5	10.2	8.9	7.6	6.5	5.9	-15.4	-42.2	-9.2	**-56.6**
18	21.5	21.9	21.1	19.8	18.3	16.5	15.2	13.0	11.5	10.7	-14.9	-35.2	-7.0	**-50.2**
19	25.3	25.8	25.0	24.6	22.3	20.9	18.9	17.0	15.6	14.4	-11.9	-31.1	-7.7	**-43.1**
**Abortion ratio^§^**														
<15	878	851	875	889	927	961	931	882	862	806	5.6	-16.1	-6.5	**-8.2**
15	618	590	555	578	570	611	582	541	505	532	-7.8	-12.9	5.3	**-13.9**
16	484	475	458	441	432	442	426	397	383	395	-10.7	-10.6	3.1	**-18.4**
17	395	385	375	368	361	362	358	331	321	320	-8.6	-11.6	-0.3	**-19.0**
18	386	377	359	359	345	351	351	328	315	320	-10.6	-8.8	1.6	**-17.1**
19	319	312	298	302	296	300	290	270	269	265	-7.2	-11.7	-1.5	**-16.9**
**Total (no.)**	**92,467**	**94,534**	**92,538**	**90,327**	**82,758**	**75,208**	**65,930**	**56,686**	**49,332**	**44,984**	**—**	**—**	**—**	**—**

* Data from 38 reporting areas; by year, these areas represent 71-80% of all abortions reported to CDC for adolescents during 2005–2014. Excludes 14 reporting areas (California, District of Columbia, Florida, Illinois, Louisiana, Maine, Maryland, Massachusetts, New Hampshire, Pennsylvania, Rhode Island, Texas, Vermont, and Wyoming) that did not report, did not report age among adolescents by individual year, or did not meet reporting standards for ≥1 year.

^†^ Number of abortions obtained by adolescents in a given age group per 1,000 adolescents in that same age group. Adolescents aged 13–14 years were used as the denominator for adolescents aged <15 years.

^§^ Number of abortions obtained by adolescents in a given age group per 1,000 live births to adolescents in that same age group.

In 2014, the abortion ratio for adolescents decreased with increasing age and was lowest among adolescents aged 19 years (Table 5). During 2005–2014, abortion ratios decreased among adolescents of all ages (Table 6).

### Gestational Age

Among the 40 areas that reported gestational age at the time of abortion for 2014, the majority (64.9%) of abortions were performed by ≤8 weeks’ gestation, and 91.0% were performed at ≤13 weeks’ gestation (Table 7). Few abortions were performed at 14–20 weeks’ gestation (7.7%) or at ≥21 weeks’ gestation (1.3%). Among the 32 reporting areas that provided data on gestational age every year during 2005–2014, the percentage of abortions performed at ≤13 weeks’ gestation was stable (Table 8). However, within this gestational age range, a shift occurred toward earlier gestational ages, with the percentage of abortions performed at ≤8 weeks’ gestation increasing 2% and the percentage of abortions performed at 9–13 weeks’ gestation decreasing 6%. For the entire period of analysis, abortions performed at >13 weeks’ gestation accounted for ≤9.1% of abortions.

**TABLE 7:** Reported abortions, by known weeks of gestation* and reporting area of occurrence — selected reporting areas,^†^ United States, 2014

State/Area	Weeks of gestation						Total abortions reported by known gestational age
	≤8	9–13	14–15	16–17	18–20	≥21	
	No. (%)^§^	No. (%)	No. (%)	No. (%)	No. (%)	No. (%)	No. (% of all reported abortions)^¶^
Alabama	4,623 (57.2)	2,657 (32.9)	407 (5.0)	207 (2.6)	180 (2.2)	6 (0.1)	**8,080 (100.0)**
Alaska	942 (62.1)	478 (31.5)	21 (1.4)	36 (2.4)	25 (1.6)	16 (1.1)	**1,518 (100.0)**
Arizona	8,380 (65.0)	3,401 (26.4)	485 (3.8)	273 (2.1)	219 (1.7)	134 (1.0)	**12,892 (99.9)**
Arkansas	2,452 (57.7)	1,186 (27.9)	247 (5.8)	167 (3.9)	168 (4.0)	33 (0.8)	**4,253 (100.0)**
Colorado	7,501 (71.6)	2,099 (20.0)	302 (2.9)	187 (1.8)	79 (0.8)	308 (2.9)	**10,476 (98.4)**
Delaware	1,843 (62.8)	973 (33.2)	79 (2.7)	27 (0.9)	—**	—	**2,933 (99.9)**
Georgia	18,245 (60.9)	8,340 (27.8)	1,059 (3.5)	748 (2.5)	824 (2.8)	739 (2.5)	**29,955 (99.8)**
Hawaii	1,252 (58.9)	667 (31.4)	64 (3.0)	71 (3.3)	51 (2.4)	20 (0.9)	**2,125 (99.0)**
Idaho	923 (68.4)	402 (29.8)	18 (1.3)	—	—	—	**1,350 (99.8)**
Indiana	4,995 (61.5)	3,082 (38.0)	19 (0.2)	—	11 (0.1)	—	**8,117 (100.0)**
Iowa	2,920 (72.7)	815 (20.3)	106 (2.6)	102 (2.5)	67 (1.7)	8 (0.2)	**4,018 (100.0)**
Kansas	4,794 (66.4)	1,786 (24.7)	203 (2.8)	190 (2.6)	187 (2.6)	59 (0.8)	**7,219 (100.0)**
Kentucky	2,123 (61.7)	934 (27.1)	147 (4.3)	101 (2.9)	88 (2.6)	49 (1.4)	**3,442 (100.0)**
Louisiana	6,848 (66.8)	2,670 (26.1)	434 (4.2)	209 (2.0)	70 (0.7)	14 (0.1)	**10,245 (99.3)**
Maine	1,308 (64.7)	576 (28.5)	68 (3.4)	28 (1.4)	28 (1.4)	13 (0.6)	**2,021 (100.0)**
Michigan	16,899 (61.2)	7,559 (27.4)	1,318 (4.8)	770 (2.8)	595 (2.2)	454 (1.6)	**27,595 (99.9)**
Minnesota	6,638 (65.6)	2,492 (24.6)	401 (4.0)	244 (2.4)	251 (2.5)	97 (1.0)	**10,123 (100.0)**
Mississippi	1,420 (61.9)	682 (29.7)	167 (7.3)	26 (1.1)	0 (0.0)	0 (0.0)	**2,295 (99.7)**
Missouri	2,849 (56.3)	1,572 (31.1)	191 (3.8)	188 (3.7)	184 (3.6)	76 (1.5)	**5,060 (100.0)**
Montana	1,127 (66.8)	405 (24.0)	64 (3.8)	37 (2.2)	46 (2.7)	9 (0.5)	**1,688 (99.9)**
Nebraska	1,540 (68.0)	576 (25.4)	76 (3.4)	55 (2.4)	—	—	**2,265 (99.8)**
Nevada	5,655 (70.3)	1,783 (22.2)	271 (3.4)	152 (1.9)	134 (1.7)	54 (0.7)	**8,049 (99.0)**
New Jersey^††^	15,210 (63.4)	4,981 (20.7)	1,292 (5.4)	956 (4.0)	849 (3.5)	720 (3.0)	**24,008 (99.3)**
New Mexico	2,845 (63.8)	875 (19.6)	155 (3.5)	109 (2.4)	124 (2.8)	352 (7.9)	**4,460 (99.1)**
New York City	45,677 (67.6)	15,083 (22.3)	2,083 (3.1)	1,441 (2.1)	1,730 (2.6)	1,574 (2.3)	**67,588 (100.0)**
North Carolina	16,454 (68.6)	5,894 (24.6)	811 (3.4)	449 (1.9)	357 (1.5)	14 (0.1)	**23,979 (97.5)**
North Dakota	788 (62.3)	424 (33.5)	41 (3.2)	11 (0.9)	0 (0.0)	0 (0.0)	**1,264 (100.0)**
Ohio	11,088 (52.3)	7,501 (35.4)	1,105 (5.2)	739 (3.5)	620 (2.9)	133 (0.6)	**21,186 (100.0)**
Oklahoma	3,692 (75.2)	1,032 (21.0)	124 (2.5)	41 (0.8)	20 (0.4)	0 (0.0)	**4,909 (99.9)**
Oregon	5,213 (65.5)	1,970 (24.8)	226 (2.8)	171 (2.2)	227 (2.9)	146 (1.8)	**7,953 (96.6)**
Rhode Island	1,970 (66.9)	710 (24.1)	128 (4.3)	55 (1.9)	65 (2.2)	18 (0.6)	**2,946 (98.5)**
South Carolina	4,018 (70.3)	1,600 (28.0)	39 (0.7)	9 (0.2)	16 (0.3)	32 (0.6)	**5,714 (100.0)**
South Dakota	329 (60.0)	211 (38.5)	0 (0.0)	—	—	7 (1.3)	**548 (99.5)**
Tennessee	7,993 (65.5)	3,786 (31.0)	364 (3.0)	15 (0.1)	17 (0.1)	24 (0.2)	**12,199 (98.6)**
Texas	33,388 (61.7)	16,306 (30.1)	2,347 (4.3)	1,253 (2.3)	603 (1.1)	235 (0.4)	**54,132 (100.0)**
Utah	2,018 (69.0)	673 (23.0)	108 (3.7)	60 (2.1)	47 (1.6)	20 (0.7)	**2,926 (99.3)**
Vermont	890 (72.1)	264 (21.4)	34 (2.8)	20 (1.6)	19 (1.5)	7 (0.6)	**1,234 (99.9)**
Virginia	15,486 (76.8)	4,307 (21.4)	117 (0.6)	96 (0.5)	112 (0.6)	47 (0.2)	**20,165 (99.9)**
Washington	12,333 (69.8)	3,828 (21.7)	490 (2.8)	335 (1.9)	322 (1.8)	363 (2.1)	**17,671 (99.8)**
West Virginia	1,078 (62.3)	546 (31.6)	55 (3.2)	25 (1.4)	19 (1.1)	6 (0.3)	**1,729 (99.9)**
**Total**	**285,747 (64.9)**	**115,126 (26.1)**	**15,666 (3.6)**	**9,611 (2.2)**	**8,385 (1.9)**	**5,795 (1.3)**	**440,330 (99.6)^§§^**

* Gestational age based on the clinician’s estimate (Alabama, Alaska, Arizona, Colorado, Delaware, Georgia, Hawaii, Idaho, Indiana, Iowa, Kansas, Kentucky, Louisiana, Maine, Michigan, Minnesota, Mississippi, Missouri, Montana, Nebraska, Nevada, New Jersey, New Mexico, New York City, North Carolina, North Dakota, Ohio, Oregon, Rhode Island, South Carolina, South Dakota, Tennessee, Utah, Vermont, Washington, and West Virginia); gestational age calculated from the last normal menstrual period (Arkansas, and Oklahoma); physician’s estimate of gestation based on estimated date of conception (Virginia); probable postfertilization age (Arkansas, and Texas). Two weeks were added to the probable postfertilization age to provide a corresponding measure to clinician’s estimate.

^†^ Data are from 40 reporting areas; excludes 12 areas (California, Connecticut, District of Columbia, Florida, Illinois, Maryland, Massachusetts, New Hampshire, New York State, Pennsylvania, Wisconsin, and Wyoming) that did not report, did not report by gestational age, or did not meet reporting standards.

^§^ Percentages for the individual component categories might not add to 100 because of rounding.

^¶^ Percentage is calculated as the number of abortions reported by known gestational age divided by the sum of abortions reported by known and unknown gestational age.

** Cell details are not displayed because of small numbers (n = 1–4).

^††^ Data from hospitals and licensed ambulatory care facilities only; because reporting is not mandatory for private physicians and women’s centers, information could not be obtained for all abortions performed in New Jersey.

^§§^ Percentage is based on 442,287 abortions reported among the areas that met reporting standards for gestational age.

**TABLE 8:** Reported abortions, by known weeks of gestation and year — selected reporting areas,* United States, 2005–2014

Weeks of gestation	Year										% change			
	2005	2006	2007	2008	2009	2010	2011	2012	2013	2014	2005 to 2009	2010 to 2014	2013 to 2014	2005 to 2014
**≤13 weeks’ gestation (%)**	**91.5**	**91.7**	**91.6**	**91.5**	**91.8**	**91.9**	**91.5**	**91.5**	**91.6**	**90.9**	**0.3**	**-1.1**	**-0.8**	**-0.7**
≤8	63.5	63.7	63.9	64.3	65.4	66.0	65.8	66.0	66.0	64.7	3.0	-2.0	-2.0	**1.9**
9–13	28.0	28.0	27.7	27.2	26.4	25.9	25.7	25.5	25.6	26.2	-5.7	1.2	2.3	**-6.4**
**>13 weeks’ gestation (%)**	**8.5**	**8.3**	**8.4**	**8.5**	**8.2**	**8.2**	**8.5**	**8.4**	**8.5**	**9.1**	**-3.5**	**11.0**	**7.1**	**7.1**
14–15	3.3	3.3	3.3	3.4	3.3	3.3	3.4	3.4	3.4	3.5	0.0	6.1	2.9	**6.1**
16–17	1.9	1.8	1.9	1.9	1.8	1.8	1.8	1.8	1.9	2.2	-5.3	22.2	15.8	**15.8**
18–20	1.9	1.9	1.9	1.9	1.8	1.8	1.9	1.9	1.9	2.0	-5.3	11.1	5.3	**5.3**
≥ 21	1.4	1.3	1.3	1.3	1.3	1.3	1.4	1.3	1.3	1.4	-7.1	7.7	7.7	**0.0**
**Total (no.)**	**516,479**	**530,739**	**523,884**	**526,547**	**502,801**	**492,341**	**466,203**	**440,764**	**419,890**	**408,342**	—	—	—	**—**

* Data from 32 reporting areas; by year, these reporting areas represent 79%–83% of the abortions reported to CDC by gestational age during 2005–2014. Excludes 20 areas (California, Connecticut, Delaware, District of Columbia, Florida, Illinois, Louisiana, Maine, Maryland, Massachusetts, Mississippi, Nebraska, Nevada, New Hampshire, New York State, Pennsylvania, Rhode Island, Vermont, Wisconsin, and Wyoming) that did not report, did not report by gestational age, or did not meet reporting standards for ≥1 year.

Among abortions performed at ≤13 weeks’ gestation and reported by individual week of gestation for 2014, 37.0% were performed at ≤6 weeks’ gestation (Table 9). The percentage contribution to abortions performed at ≤13 weeks’ gestation was progressively smaller for each additional week of gestation: 19.4% were performed at 7 weeks’ gestation, and 3.1% were performed at 13 weeks’ gestation. Among the 32 areas that reported by exact week of gestation for abortions performed at ≤13 weeks’ gestation every year during 2005–2014, a shift occurred toward the earliest gestational age reported: abortions performed at ≤6 weeks’ gestation increased 9.1%, and those performed at 7–13 weeks’ gestation decreased 0%–12% (Table 10).

**TABLE 9:** Reported abortions obtained at ≤13 weeks’ gestation,* by weeks of gestation and reporting area of occurrence — selected reporting areas,^†^ United States, 2014

State/Area	Weeks of gestation								Total no. of abortions at ≤13weeks
	≤6	7	8	9	10	11	12	13	
	No. (%)^§^	No. (%)	No. (%)	No. (%)	No. (%)	No. (%)	No. (%)	No. (%)	
Alabama	1,680 (23.1)	1,689 (23.2)	1,254 (17.2)	818 (11.2)	650 (8.9)	489 (6.7)	387 (5.3)	313 (4.3)	**7,280**
Alaska	436 (30.7)	278 (19.6)	228 (16.1)	133 (9.4)	98 (6.9)	78 (5.5)	91 (6.4)	78 (5.5)	**1,420**
Arizona	3,672 (31.2)	2,813 (23.9)	1,895 (16.1)	1,103 (9.4)	859 (7.3)	676 (5.7)	351 (3.0)	412 (3.5)	**11,781**
Arkansas	1,159 (31.9)	627 (17.2)	666 (18.3)	370 (10.2)	256 (7.0)	270 (7.4)	130 (3.6)	160 (4.4)	**3,638**
Colorado	4,289 (44.7)	1,917 (20.0)	1,295 (13.5)	770 (8.0)	456 (4.8)	408 (4.3)	243 (2.5)	222 (2.3)	**9,600**
Delaware	764 (27.1)	567 (20.1)	512 (18.2)	369 (13.1)	202 (7.2)	172 (6.1)	120 (4.3)	110 (3.9)	**2,816**
Georgia	8,491 (31.9)	5,740 (21.6)	4,014 (15.1)	2,555 (9.6)	1,744 (6.6)	1,598 (6.0)	1,421 (5.3)	1,022 (3.8)	**26,585**
Hawaii	607 (31.6)	343 (17.9)	302 (15.7)	230 (12.0)	119 (6.2)	110 (5.7)	113 (5.9)	95 (5.0)	**1,919**
Idaho	358 (27.0)	326 (24.6)	239 (18.0)	148 (11.2)	90 (6.8)	58 (4.4)	57 (4.3)	49 (3.7)	**1,325**
Indiana	1,720 (21.3)	1,675 (20.7)	1,600 (19.8)	1,001 (12.4)	715 (8.9)	589 (7.3)	399 (4.9)	378 (4.7)	**8,077**
Iowa	1,551 (41.5)	770 (20.6)	599 (16.0)	262 (7.0)	179 (4.8)	146 (3.9)	147 (3.9)	81 (2.2)	**3,735**
Kansas	2,528 (38.4)	1,245 (18.9)	1,021 (15.5)	667 (10.1)	422 (6.4)	304 (4.6)	246 (3.7)	147 (2.2)	**6,580**
Kentucky	733 (24.0)	760 (24.9)	630 (20.6)	317 (10.4)	230 (7.5)	162 (5.3)	148 (4.8)	77 (2.5)	**3,057**
Louisiana	3,563 (37.4)	1,864 (19.6)	1,421 (14.9)	932 (9.8)	596 (6.3)	429 (4.5)	358 (3.8)	355 (3.7)	**9,518**
Maine	487 (25.8)	467 (24.8)	354 (18.8)	200 (10.6)	107 (5.7)	99 (5.3)	93 (4.9)	77 (4.1)	**1,884**
Michigan	8,128 (33.2)	4,794 (19.6)	3,977 (16.3)	2,604 (10.6)	1,592 (6.5)	1,327 (5.4)	1,201 (4.9)	835 (3.4)	**24,458**
Minnesota	3,281 (35.9)	1,956 (21.4)	1,401 (15.3)	908 (9.9)	558 (6.1)	449 (4.9)	294 (3.2)	283 (3.1)	**9,130**
Mississippi	507 (24.1)	570 (27.1)	343 (16.3)	214 (10.2)	178 (8.5)	141 (6.7)	84 (4.0)	65 (3.1)	**2,102**
Missouri	1,125 (25.4)	947 (21.4)	777 (17.6)	517 (11.7)	381 (8.6)	346 (7.8)	210 (4.8)	118 (2.7)	**4,421**
Montana	624 (40.7)	267 (17.4)	236 (15.4)	110 (7.2)	74 (4.8)	80 (5.2)	76 (5.0)	65 (4.2)	**1,532**
Nebraska	985 (46.6)	303 (14.3)	252 (11.9)	176 (8.3)	120 (5.7)	101 (4.8)	106 (5.0)	73 (3.4)	**2,116**
Nevada	2,927 (39.4)	1,609 (21.6)	1,119 (15.0)	733 (9.9)	394 (5.3)	320 (4.3)	204 (2.7)	132 (1.8)	**7,438**
New Jersey^¶^	8,476 (42.0)	4,071 (20.2)	2,663 (13.2)	1,650 (8.2)	1,092 (5.4)	624 (3.1)	765 (3.8)	850 (4.2)	**20,191**
New Mexico	1,825 (49.1)	596 (16.0)	424 (11.4)	276 (7.4)	230 (6.2)	147 (4.0)	143 (3.8)	79 (2.1)	**3,720**
New York City	25,469 (41.9)	11,580 (19.1)	8,628 (14.2)	5,723 (9.4)	3,474 (5.7)	2,634 (4.3)	2,042 (3.4)	1,210 (2.0)	**60,760**
North Carolina	8,649 (38.7)	4,455 (19.9)	3,350 (15.0)	1,967 (8.8)	1,365 (6.1)	1,049 (4.7)	866 (3.9)	647 (2.9)	**22,348**
North Dakota	349 (28.8)	251 (20.7)	188 (15.5)	164 (13.5)	80 (6.6)	90 (7.4)	53 (4.4)	37 (3.1)	**1,212**
Ohio	4,584 (24.7)	3,516 (18.9)	2,988 (16.1)	2,378 (12.8)	1,704 (9.2)	1,435 (7.7)	1,107 (6.0)	877 (4.7)	**18,589**
Oklahoma	2,698 (57.1)	542 (11.5)	452 (9.6)	351 (7.4)	200 (4.2)	226 (4.8)	156 (3.3)	99 (2.1)	**4,724**
Oregon	2,768 (38.5)	1,340 (18.7)	1,105 (15.4)	630 (8.8)	449 (6.3)	344 (4.8)	323 (4.5)	224 (3.1)	**7,183**
Rhode Island	1,153 (43.0)	494 (18.4)	323 (12.1)	254 (9.5)	148 (5.5)	125 (4.7)	87 (3.2)	96 (3.6)	**2,680**
South Carolina	2,009 (35.8)	1,075 (19.1)	934 (16.6)	505 (9.0)	394 (7.0)	386 (6.9)	156 (2.8)	159 (2.8)	**5,618**
South Dakota	134 (24.8)	122 (22.6)	73 (13.5)	69 (12.8)	44 (8.1)	42 (7.8)	26 (4.8)	30 (5.6)	**540**
Tennessee	4,033 (34.2)	2,199 (18.7)	1,761 (15.0)	1,236 (10.5)	878 (7.5)	816 (6.9)	454 (3.9)	402 (3.4)	**11,779**
Texas	17,650 (35.5)	8,634 (17.4)	7,104 (14.3)	5,634 (11.3)	3,825 (7.7)	3,238 (6.5)	1,873 (3.8)	1,736 (3.5)	**49,694**
Utah	1,006 (37.4)	590 (21.9)	422 (15.7)	208 (7.7)	175 (6.5)	104 (3.9)	64 (2.4)	122 (4.5)	**2,691**
Vermont	405 (35.1)	274 (23.7)	211 (18.3)	90 (7.8)	57 (4.9)	47 (4.1)	36 (3.1)	34 (2.9)	**1,154**
Virginia	10,227 (51.7)	2,920 (14.8)	2,339 (11.8)	1,594 (8.1)	1,125 (5.7)	866 (4.4)	466 (2.4)	256 (1.3)	**19,793**
Washington	6,869 (42.5)	3,104 (19.2)	2,360 (14.6)	1,172 (7.3)	904 (5.6)	743 (4.6)	510 (3.2)	499 (3.1)	**16,161**
West Virginia	471 (29.0)	334 (20.6)	273 (16.8)	160 (9.9)	123 (7.6)	118 (7.3)	105 (6.5)	40 (2.5)	**1,624**
**Total**	**148,390 (37.0)**	**77,624 (19.4)**	**59,733 (14.9)**	**39,198 (9.8)**	**26,287 (6.6)**	**21,386 (5.3)**	**15,711 (3.9)**	**12,544 (3.1)**	**400,873**

* Gestational age based on the clinician’s estimate (Alabama, Alaska, Arizona, Colorado, Delaware, Georgia, Hawaii, Idaho, Indiana, Iowa, Kansas, Kentucky, Louisiana, Maine, Michigan, Minnesota, Mississippi, Missouri, Montana, Nebraska, Nevada, New Jersey, New Mexico, New York City, North Carolina, North Dakota, Ohio, Oregon, Rhode Island, South Carolina, South Dakota, Tennessee, Utah, Vermont, Washington, and West Virginia); gestational age calculated from the last normal menstrual period (Arkansas and Oklahoma); physician’s estimate of gestation based on estimated date of conception (Virginia); probable postfertilization age (Arkansas and Texas). Two weeks were added to the probable postfertilization age to provide a corresponding measure to clinician’s estimate.

^†^ Data are from 40 reporting areas; excludes 12 areas (California, Connecticut, District of Columbia, Florida, Illinois, Maryland, Massachusetts, New Hampshire, New York State, Pennsylvania, Wisconsin, and Wyoming) that did not report, did not report by gestational age, or did not meet reporting standards.

^§^ Percentages for the individual component categories might not add to 100 because of rounding.

^¶^ Data from hospitals and licensed ambulatory care facilities only; because reporting is not mandatory for private physicians and women’s centers, information could not be obtained for all abortions performed in New Jersey.

**TABLE 10:** Reported abortions obtained at ≤13 weeks’ gestation, by weeks of gestation and year — selected reporting areas,* United States, 2005–2014

Weeks of gestation	Year										% change			
	2005	2006	2007	2008	2009	2010	2011	2012	2013	2014	2005 to 2009	2010 to 2014	2013 to 2014	2005 to 2014
**% distribution among abortions reported at ≤13 weeks**														
≤6	34.0	34.1	35.1	35.6	36.9	37.9	37.7	38.5	37.9	37.1	8.5	-2.1	-2.1	**9.1**
7	19.8	20.1	20.0	19.9	19.5	19.3	19.6	19.3	19.5	19.3	-1.5	0.0	-1.0	**-2.5**
8	15.6	15.2	14.7	14.8	14.9	14.5	14.6	14.3	14.6	14.9	-4.5	2.8	2.1	**-4.5**
9	10.4	10.4	10.2	10.0	9.7	9.7	9.5	9.4	9.4	9.8	-6.7	1.0	4.3	**-5.8**
10	7.5	7.4	7.3	7.1	6.8	6.6	6.5	6.3	6.4	6.6	-9.3	0.0	3.1	**-12.0**
11	5.4	5.4	5.4	5.5	5.3	5.1	5.2	5.1	5.1	5.4	-1.9	5.9	5.9	**0.0**
12	4.2	4.3	4.2	4.2	4.1	3.9	4.0	3.9	4.0	3.9	-2.4	0.0	-2.5	**-7.1**
13	3.1	3.1	3.1	3.0	2.9	2.8	2.9	3.0	3.1	3.1	-6.5	10.7	0.0	**0.0**
**Total (no.)**	**472,645**	**486,347**	**479,817**	**481,887**	**461,904**	**452,476**	**426,687**	**403,275**	**384,371**	**371,165**	—	—	—	—

* Data from 32 reporting areas; by year, these reporting areas represent 84%–90% of the abortions reported to CDC at ≤13 weeks’ gestation during 2005–2014. Excludes 20 reporting areas (California, Connecticut, Delaware, District of Columbia, Florida, Illinois, Louisiana, Maine, Maryland, Massachusetts, Mississippi, Nebraska, Nevada, New Hampshire, New York State, Pennsylvania, Rhode Island, Vermont, Wisconsin, and Wyoming) that did not report, did not report by gestational age, or did not meet reporting standards for ≥1 year.

### Method Type

Among the 43 areas that reported by method type for 2014 and included medical abortion on their reporting form for medical providers, 66.9% of abortions were surgical abortions at ≤13 weeks’ gestation, 22.5% were early medical abortions (a nonsurgical abortion at ≤8 weeks’ gestation), and 9.1% were surgical abortions at >13 weeks’ gestation; all other methods were uncommon (<1.5%) (Table 11). Among the 35 reporting areas that included medical abortion on their reporting form and provided these data for the relevant years of comparison (2005 versus 2014, 2005 versus 2009, 2010 versus 2014, and 2013 versus 2014),^§§§^ use of early medical abortion increased 1% from 2013 to 2014 (from 22.2% of abortions to 22.5%); from 2005 to 2014, use of early medical abortion increased 110% (from 10.7% of abortions to 22.5%). Increases in early medical abortion occurred both from 2005 to 2009 (from 10.7% of abortions to 16.5% [54% increase]) and from 2010 to 2014 (from 18.4% of abortions to 22.5% [22% increase]).

**TABLE 11:** Reported abortions, by known method type and reporting area of occurrence — selected reporting areas,* United States, 2014

State/Area	Surgical^†^			Medical			Intrauterine instillation^§^	Hysterectomy/ Hysterotomy	Total abortions reported by known method type
	Surgical, ≤13 weeks’ gestation	Surgical, >13 weeks’ gestation	Surgical, unknown gestational age	Medical, ≤8 weeks’ gestation	Medical, >8 weeks’ gestation	Medical, unknown gestational age			
	No. (%)^¶^	No. (%)	No. (%)	No. (%)	No. (%)	No. (%)	No. (%)	No. (%)	No. (% of all reported abortions)**
Alabama	5,280 (65.5)	778 (9.6)	0 (0.0)	1,901 (23.6)	104 (1.3)	0 (0.0)	—^††^	—	**8,067 (99.8)**
Alaska	1,122 (74.3)	93 (6.2)	0 (0.0)	288 (19.1)	6 (0.4)	0 (0.0)	—	—	**1,511 (99.5)**
Arizona	8,213 (63.7)	1,078 (8.4)	6 (0.0)	3,469 (26.9)	119 (0.9)	—	6 (0.0)	—	**12,893 (99.9)**
Arkansas	3,030 (71.2)	612 (14.4)	0 (0.0)	547 (12.9)	61 (1.4)	0 (0.0)	—	—	**4,253 (100.0)**
Colorado	5,204 (50.2)	574 (5.5)	75 (0.7)	4,210 (40.6)	196 (1.9)	96 (0.9)	—	—	**10,357 (97.3)**
Connecticut^§§^	NA	NA	6,296 (59.3)	NA	NA	4,314 (40.7)	—	—	**10,611 (100.0)**
Delaware	1,522 (51.9)	114 (3.9)	—	1,190 (40.6)	102 (3.5)	0 (0.0)	—	0 (0.0)	**2,932 (99.8)**
District of Columbia^¶¶^	1,409 (50.5)	472 (16.9)	0 (0.0)	909 (32.6)	0 (0.0)	0 (0.0)	0 (0.0)	0 (0.0)	**2,790 (100.0)**
Georgia	20,652 (69.3)	3,363 (11.3)	—	5,629 (18.9)	159 (0.5)	—	—	9 (0.0)	**29,818 (99.4)**
Idaho	779 (57.6)	21 (1.6)	—	523 (38.7)	24 (1.8)	0 (0.0)	—	—	**1,352 (99.9)**
Indiana	5,912 (72.8)	40 (0.5)	—	2,062 (25.4)	103 (1.3)	0 (0.0)	—	0 (0.0)	**8,118 (100.0)**
Iowa	1,571 (39.3)	268 (6.7)	—	2,072 (51.8)	88 (2.2)	—	—	0 (0.0)	**4,001 (99.5)**
Kansas	3,352 (46.4)	638 (8.8)	0 (0.0)	3,042 (42.2)	185 (2.6)	0 (0.0)	0 (0.0)	0 (0.0)	**7,217 (100.0)**
Kentucky	1,959 (56.9)	377 (11.0)	0 (0.0)	1,093 (31.8)	11 (0.3)	0 (0.0)	0 (0.0)	0 (0.0)	**3,440 (99.9)**
Maine	1,323 (65.5)	103 (5.1)	0 (0.0)	522 (25.8)	72 (3.6)	0 (0.0)	0 (0.0)	0 (0.0)	**2,020 (100.0)**
Massachusetts^§§^	NA	NA	14,191 (73.9)	NA	NA	5,013 (26.1)	—	—	**19,206 (99.2)**
Michigan	17,571 (63.6)	3,075 (11.1)	28 (0.1)	6,451 (23.4)	478 (1.7)	6 (0.0)	5 (0.0)	0 (0.0)	**27,614 (99.9)**
Minnesota	6,285 (62.1)	966 (9.5)	0 (0.0)	2,650 (26.2)	204 (2.0)	—	15 (0.1)	—	**10,121 (100.0)**
Mississippi	1,296 (56.3)	188 (8.2)	—	746 (32.4)	65 (2.8)	7 (0.3)	0 (0.0)	—	**2,303 (100.0)**
Missouri	3,259 (64.4)	631 (12.5)	0 (0.0)	1,141 (22.6)	27 (0.5)	0 (0.0)	—	—	**5,059 (100.0)**
Montana	671 (39.7)	151 (8.9)	0 (0.0)	835 (49.4)	31 (1.8)	—	—	0 (0.0)	**1,690 (100.0)**
Nebraska	1,157 (51.0)	148 (6.5)	—	929 (41.0)	28 (1.2)	3 (0.1)	—	—	**2,267 (99.9)**
Nevada	5,618 (71.8)	609 (7.8)	46 (0.6)	1,469 (18.8)	44 (0.6)	34 (0.4)	0 (0.0)	0 (0.0)	**7,820 (96.2)**
New Jersey***	15,592 (64.5)	3,783 (15.6)	64 (0.3)	4,377 (18.1)	232 (1.0)	106 (0.4)	—	—	**24,181 (100.0)**
New Mexico	2,520 (59.1)	460 (10.8)	18 (0.4)	972 (22.8)	282 (6.6)	8 (0.2)	—	—	**4,261 (94.7)**
New York	63,539 (66.4)	8,314 (8.7)	5,205 (5.4)	14,716 (15.4)	2,425 (2.5)	1,437 (1.5)	90 (0.1)	12 (0.0)	**95,738 (99.0)**
New York City	49,646 (73.6)	6,490 (9.6)	23 (0.0)	10,533 (15.6)	692 (1.0)	9 (0.0)	62 (0.1)	12 (0.0)	**67,467 (99.8)**
New York State	13,893 (49.1)	1,824 (6.5)	5,182 (18.3)	4,183 (14.8)	1,733 (6.1)	1,428 (5.1)	28 (0.1)	0 (0.0)	**28,271 (97.2)**
North Carolina	13,212 (56.4)	1,574 (6.7)	252 (1.1)	7,783 (33.2)	269 (1.1)	331 (1.4)	0 (0.0)	0 (0.0)	**23,421 (95.2)**
North Dakota	1,078 (85.5)	47 (3.7)	0 (0.0)	111 (8.8)	25 (2.0)	0 (0.0)	0 (0.0)	0 (0.0)	**1,261 (99.8)**
Ohio	17,519 (82.8)	2,569 (12.1)	0 (0.0)	1,039 (4.9)	23 (0.1)	0 (0.0)	0 (0.0)	5 (0.0)	**21,155 (99.9)**
Oklahoma	2,907 (59.3)	181 (3.7)	—	1,806 (36.8)	—	—	—	—	**4,906 (99.8)**
Oregon	4,841 (59.1)	722 (8.8)	184 (2.2)	2,241 (27.3)	111 (1.4)	92 (1.1)	—	—	**8,196 (99.6)**
Pennsylvania	18,256 (56.9)	4,053 (12.6)	0 (0.0)	9,133 (28.4)	666 (2.1)	0 (0.0)	—	—	**32,111 (100.0)**
Rhode Island	1,875 (63.0)	256 (8.6)	11 (0.4)	733 (24.6)	67 (2.3)	32 (1.1)	—	—	**2,975 (99.5)**
South Carolina	3,195 (55.9)	64 (1.1)	0 (0.0)	2,331 (40.8)	117 (2.0)	0 (0.0)	—	—	**5,712 (100.0)**
South Dakota	316 (57.4)	0 (0.0)	—	196 (35.6)	35 (6.4)	—	0 (0.0)	—	**551 (100.0)**
Texas	44,731 (82.6)	4,366 (8.1)	15 (0.0)	4,928 (9.1)	92 (0.2)	—	—	—	**54,137 (100.0)**
Utah	1,806 (61.7)	213 (7.3)	8 (0.3)	852 (29.1)	37 (1.3)	13 (0.4)	0 (0.0)	0 (0.0)	**2,929 (99.4)**
Vermont	631 (51.3)	67 (5.4)	0 (0.0)	506 (41.1)	26 (2.1)	—	—	0 (0.0)	**1,231 (99.7)**
Virginia	14,858 (73.7)	347 (1.7)	18 (0.1)	4,848 (24.1)	72 (0.4)	—	—	0 (0.0)	**20,147 (99.8)**
Washington	10,671 (60.3)	1,494 (8.4)	14 (0.1)	5,397 (30.5)	101 (0.6)	25 (0.1)	—	—	**17,705 (100.0)**
West Virginia	1,345 (77.7)	88 (5.1)	—	275 (15.9)	21 (1.2)	0 (0.0)	0 (0.0)	—	**1,730 (100.0)**
Wisconsin^§§,†††^	NA	NA	4,554 (80.7)	NA	NA	1,086 (19.3)	0 (0.0)	0 (0.0)	**5,640 (97.2)**
**Total**	**343,376 (66.9)**	**46,602 (9.1)**	—**^§§§^**	**115,777 (22.5)**	**7,477 (1.5)**	—**^¶¶¶^**	**153 (0.0)**	**62 (0.0)**	513,447 (99.3)****

**Abbreviation:** NA = not available.

* Data from 43 reporting areas; excludes nine reporting areas (California, Florida, Hawaii, Illinois, Louisiana, Maryland, New Hampshire, Tennessee, and Wyoming) that did not report, did not report by method type, or did not meet reporting standards.

^†^ Includes aspiration curettage, suction curettage, manual vacuum aspiration, menstrual extraction, sharp curettage, and dilation and evacuation procedures.

^§^ Intrauterine instillations reported at ≤12 weeks’ gestation are not presented with abortions reported by known method type.

^¶^ Percentages for the individual component categories might not add to 100 because of rounding and because some areas report more than one method for each abortion.

** Percentage is calculated as the number of abortions reported by known method type divided by the sum of abortions reported by known and unknown method type.

^††^ Cell details are not displayed because of small numbers (n = 1–4).

^§§^ Numbers for surgical procedures at ≤13 weeks versus >13 weeks and for medical abortion at ≤8 weeks versus >8 weeks are not presented because gestational age data were not provided or were provided in incompatible categories.

^¶¶^ Because reporting is not mandatory, a complete number of abortions performed in the District of Columbia could not be obtained.

*** Data from hospitals and licensed ambulatory care facilities only; because reporting is not mandatory for private physicians and women’s centers, information could not be obtained for all abortions performed in New Jersey.

^†††^ All abortions were reported as surgical or chemically induced. For this report, all surgical abortions were classified as surgical and all chemical abortions as medical.

^§§§^ Surgical abortions reported without a gestational age were distributed among the surgical abortion categories according to the distribution of surgical abortions at known gestational age.

^¶¶¶^ Medical abortions reported without a gestational age were distributed among the medical abortion categories according to the distribution of medical abortions at known gestational age.

**** Percentage is based on 517,217 abortions reported among the areas that met reporting standards for method type.

Among the 31 reporting areas that provided data by procedure and individual week of gestational age each year from 2011 to 2014, the percentage of abortions at 9 weeks’ gestation that were medical abortions did not change substantially between 2011, 2012, and 2013 (5.0%, 5.8%, and 6.8%, respectively), but then increased to 7.7% in 2014.

In contrast to the increase that occurred in use of early medical abortion, use of surgical abortion at ≤13 weeks’ gestation decreased 15% from 2005 to 2014 (from 78.8% of abortions to 66.9%). Surgical abortion at >13 weeks’ gestation consistently accounted for approximately 8.0–9.0% of all abortions, and all other methods consistently accounted for a small percentage of abortions (<0.1%–1.3%) during 2005–2014.

### Race/Ethnicity

Among the 30 areas that reported cross-classified race/ethnicity data for 2014, non-Hispanic white women and non-Hispanic black women accounted for the largest percentages of all abortions (38.0% and 36.0%, respectively), and Hispanic women and non-Hispanic women in the other race category accounted for smaller percentages (18.3% and 7.7%, respectively) (Table 12). Non-Hispanic white women had the lowest abortion rate (7.5 abortions per 1,000 women aged 15–44 years) and ratio (124 abortions per 1,000 live births) and non-Hispanic black women had the highest abortion rate (26.6 abortions per 1,000 women aged 15–44 years) and ratio (417 abortions per 1,000 live births). Data for 2014 are also reported separately by race and by ethnicity (Tables 13 and 14).

**TABLE 12:** Reported abortions, by known race/ethnicity of women who obtained an abortion and reporting area of occurrence — selected reporting areas,* United States, 2014

State/Area	Non-Hispanic			Hispanic	Total abortions reported by known race/ethnicity
	White	Black	Other		No. (% of all reported abortions)^§^
	No. (%)^†^	No. (%)	No. (%)	No. (%)	
Alabama	2,639 (32.8)	4,792 (59.6)	278 (3.5)	331 (4.1)	**8,040 (99.5)**
Arizona	5,624 (45.5)	987 (8.0)	1,191 (9.6)	4,562 (36.9)	**12,364 (95.8)**
Arkansas	1,908 (44.9)	1,949 (45.9)	165 (3.9)	226 (5.3)	**4,248 (99.9)**
Colorado	5,589 (59.5)	634 (6.8)	721 (7.7)	2,442 (26.0)	**9,386 (88.1)**
Delaware	1,195 (40.7)	1,230 (41.9)	156 (5.3)	355 (12.1)	**2,936 (100.0)**
District of Columbia^¶^	653 (23.7)	1,552 (56.3)	237 (8.6)	315 (11.4)	**2,757 (98.8)**
Georgia	6,991 (26.1)	16,985 (63.5)	1,464 (5.5)	1,323 (4.9)	**26,763 (89.2)**
Hawaii	439 (23.4)	75 (4.0)	1,138 (60.6)	226 (12.0)	**1,878 (87.5)**
Idaho	1,009 (77.4)	19 (1.5)	66 (5.1)	209 (16.0)	**1,303 (96.3)**
Indiana	4,573 (57.3)	2,284 (28.6)	520 (6.5)	601 (7.5)	**7,978 (98.3)**
Kansas	4,141 (57.7)	1,528 (21.3)	660 (9.2)	845 (11.8)	**7,174 (99.4)**
Michigan	11,806 (43.1)	13,526 (49.4)	1,029 (3.8)	1,018 (3.7)	**27,379 (99.1)**
Minnesota	5,046 (55.2)	2,153 (23.6)	1,335 (14.6)	606 (6.6)	**9,140 (90.3)**
Missouri	2,483 (49.8)	2,097 (42.1)	264 (5.3)	139 (2.8)	**4,983 (98.5)**
Montana	1,462 (86.5)	15 (0.9)	134 (7.9)	79 (4.7)	**1,690 (100.0)**
Nevada	3,123 (43.9)	1,273 (17.9)	520 (7.3)	2,201 (30.9)	**7,117 (87.5)**
New Jersey**	7,782 (35.0)	6,074 (27.3)	3,972 (17.9)	4,415 (19.8)	**22,243 (92.0)**
New York	22,532 (25.2)	34,720 (38.8)	7,659 (8.6)	24,588 (27.5)	**89,499 (92.5)**
New York City^††^	9,401 (14.7)	27,367 (42.7)	7,024 (10.9)	20,371 (31.7)	**64,163 (94.9)**
New York State	13,131 (51.8)	7,353 (29.0)	635 (2.5)	4,217 (16.6)	**25,336 (87.1)**
North Carolina	8,409 (37.5)	10,668 (47.6)	981 (4.4)	2,363 (10.5)	**22,421 (91.1)**
Ohio	9,792 (51.2)	7,487 (39.1)	1,062 (5.6)	794 (4.1)	**19,135 (90.3)**
Oregon	5,584 (72.4)	450 (5.8)	683 (8.9)	992 (12.9)	**7,709 (93.7)**
South Carolina	2,972 (52.1)	2,255 (39.5)	243 (4.3)	238 (4.2)	**5,708 (99.9)**
South Dakota	380 (69.0)	56 (10.2)	79 (14.3)	36 (6.5)	**551 (100.0)**
Tennessee	5,462 (45.6)	5,660 (47.2)	350 (2.9)	513 (4.3)	**11,985 (96.9)**
Texas^§§^	15,825 (29.4)	14,486 (26.9)	3,578 (6.6)	20,004 (37.1)	**53,893 (99.5)**
Utah	1,945 (68.8)	90 (3.2)	249 (8.8)	542 (19.2)	**2,826 (95.9)**
Vermont	1,103 (91.2)	23 (1.9)	53 (4.4)	30 (2.5)	**1,209 (97.9)**
Virginia	7,413 (38.2)	8,572 (44.2)	1,357 (7.0)	2,061 (10.6)	**19,403 (96.1)**
West Virginia	1,497 (86.5)	185 (10.7)	39 (2.3)	9 (0.5)	**1,730** (**100.0)**
**Total**	**149,377 (38.0)**	**141,825 (36.0)**	**30,183 (7.7)**	**72,063 (18.3)**	**393,448** **(94.4)**^¶¶^
Abortion rate***	**7.5**	**26.6**	**13.5**	**12.3**	**11.8**
**Abortion ratio** ^†††^	**124**	**417**	**219**	**161**	**185**

* Data from 30 reporting areas; excludes 22 reporting areas (Alaska, California, Connecticut, Florida, Illinois, Iowa, Kentucky, Louisiana, Maine, Maryland, Massachusetts, Mississippi, Nebraska, New Hampshire, New Mexico, North Dakota, Oklahoma, Pennsylvania, Rhode Island, Washington, Wisconsin, and Wyoming) that did not report, did not report by race/ethnicity, or did not meet reporting standards.

^†^ Percentages for the individual component categories might not add to 100 because of rounding.

^§^ Percentage is calculated as the number of abortions reported by known race/ethnicity divided by the sum of abortions reported by known and unknown race/ethnicity.

^¶^ Because reporting is not mandatory, a complete number of abortions performed in the District of Columbia could not be obtained.

** Data from hospitals and licensed ambulatory care facilities only; because reporting is not mandatory for private physicians and women’s centers, information could not be obtained for all abortions performed in New Jersey.

^††^ Non-Hispanic categories include abortions for women whose ethnicity was reported as unknown; previous evaluation has shown that most reports without ethnicity are for non-Hispanic women.

^§§^ Reporting form contains only one question for race and ethnicity; therefore, abortions reported for women of white, black, and other races (Asian and Native American) are not explicitly identified as non-Hispanic.

^¶¶^ Percentage is based on 416,892 abortions reported among the areas that met reporting standards for race/ethnicity.

*** Number of abortions obtained by women in a given race/ethnicity group per 1,000 women in that same group. For each reporting area, abortions for women of unknown race/ethnicity were distributed according to the distribution of abortions among women of known race/ethnicity for that area.

^†††^ Number of abortions obtained by women in a given race/ethnicity group per 1,000 live births to women in that same race/ethnicity group. For each reporting area, abortions for women of unknown race/ethnicity were distributed according to the distribution of abortions among women of known race/ethnicity for that area.

**TABLE 13:** Reported abortions, by known race of women who obtained an abortion and reporting area of occurrence — selected reporting areas,* United States, 2014

State/Area	Race			Total abortions reported by known race
	White	Black	Other	
	No. (%)^†^	No. (%)	No. (%)	No. (% of all reported abortions)^§^
Alabama	2,928 (36.3)	4,833 (59.9)	302 (3.7)	**8,063 (99.8)**
Alaska	819 (58.3)	116 (8.3)	469 (33.4)	**1,404 (92.5)**
Arizona	8,982 (78.6)	1,034 (9.1)	1,405 (12.3)	**11,421 (88.5)**
Arkansas	1,960 (46.1)	1,951 (45.9)	342 (8.0)	**4,253 (100.0)**
Colorado	6,570 (68.5)	731 (7.6)	2,285 (23.8)	**9,586 (90.0)**
Delaware	1,478 (50.3)	1,290 (43.9)	169 (5.8)	**2,937 (100.0)**
District of Columbia^¶^	823 (29.8)	1,579 (57.2)	357 (12.9)	**2,759 (98.9)**
Georgia	7,758 (28.5)	17,824 (65.4)	1,660 (6.1)	**27,242 (90.8)**
Hawaii	568 (29.0)	82 (4.2)	1,307 (66.8)	**1,957 (91.2)**
Idaho	1,150 (89.9)	22 (1.7)	107 (8.4)	**1,279 (94.5)**
Indiana	4,738 (59.1)	2,327 (29.0)	950 (11.9)	**8,015 (98.7)**
Iowa	2,983 (76.4)	534 (13.7)	386 (9.9)	**3,903 (97.1)**
Kansas	4,402 (61.0)	1,572 (21.8)	1,245 (17.2)	**7,219 (100.0)**
Louisiana	3,121 (30.4)	6,249 (60.9)	897 (8.7)	**10,267 (99.5)**
Maine	1,782 (88.3)	92 (4.6)	145 (7.2)	**2,019 (99.9)**
Massachusetts	9,011 (52.6)	3,182 (18.6)	4,933 (28.8)	**17,126 (88.5)**
Michigan	12,347 (45.7)	13,593 (50.3)	1,095 (4.1)	**27,035 (97.9)**
Minnesota	5,336 (56.1)	2,295 (24.1)	1,873 (19.7)	**9,504 (93.9)**
Mississippi	376 (17.0)	1,813 (82.0)	21 (1.0)	**2,210 (96.0)**
Missouri	2,604 (52.0)	2,123 (42.4)	280 (5.6)	**5,007 (99.0)**
Montana	1,535 (90.8)	17 (1.0)	138 (8.2)	**1,690 (100.0)**
Nebraska	1,443 (66.3)	385 (17.7)	350 (16.1)	**2,178 (95.9)**
New Jersey**	9,816 (42.6)	8,303 (36.0)	4,949 (21.5)	**23,068 (95.4)**
North Carolina	9,459 (42.8)	11,559 (52.3)	1,090 (4.9)	**22,108 (89.9)**
North Dakota	905 (73.0)	93 (7.5)	242 (19.5)	**1,240 (98.1)**
Ohio	10,775 (53.2)	8,253 (40.7)	1,233 (6.1)	**20,261 (95.6)**
Oklahoma	2,986 (60.8)	851 (17.3)	1,074 (21.9)	**4,911 (99.9)**
Oregon	6,333 (82.0)	480 (6.2)	914 (11.8)	**7,727 (93.9)**
Pennsylvania	15,691 (49.4)	13,782 (43.4)	2,306 (7.3)	**31,779 (98.9)**
Rhode Island	2,189 (76.7)	463 (16.2)	202 (7.1)	**2,854 (95.5)**
South Carolina	3,202 (56.1)	2,265 (39.7)	243 (4.3)	**5,710 (99.9)**
South Dakota	410 (74.4)	58 (10.5)	83 (15.1)	**551 (100.0)**
Tennessee	5,913 (49.3)	5,673 (47.3)	406 (3.4)	**11,992 (96.9)**
Vermont	1,128 (92.6)	26 (2.1)	64 (5.3)	**1,218 (98.6)**
Virginia	9,296 (48.0)	8,693 (44.9)	1,385 (7.1)	**19,374 (96.0)**
West Virginia	1,500 (86.7)	185 (10.7)	45 (2.6)	**1,730** (**100.0)**
Wisconsin^††^	3,579 (66.5)	1,439 (26.7)	365 (6.8)	**5,383 (95.4)**
**Total**	**165,896 (50.7)**	**125,767 (38.5)**	**35,317 (10.8)**	**326,980 (95.1)^§§^**
**Rate^¶¶^**	**6.9**	**23.2**	**16.6**	**10.3**
Ratio***	**112**	**345**	**271**	**165**

* Data from 37 reporting areas; excludes 15 areas (California, Connecticut, Florida, Illinois, Kentucky, Maryland, Nevada, New Hampshire, New Mexico, New York State, New York City, Texas, Utah, Washington, and Wyoming) that did not report, did not report by race, or did not meet reporting standards.

^†^ Percentages for the individual component categories might not add to 100 because of rounding.

^§^ Percentage is calculated as the number of abortions reported by known race, divided by the sum of abortions reported by known and unknown race.

^¶^ Because reporting is not mandatory, a complete number of abortions performed in the District of Columbia could not be obtained.

** Data from hospitals and licensed ambulatory care facilities only; because reporting is not mandatory for private physicians and women’s centers, information could not be obtained for all abortions performed in New Jersey.

^††^ Includes residents only.

^§§^ Percentage is based on 343,697 abortions reported among the areas that met reporting standards for race.

^¶¶^ Number of abortions obtained by women in a given racial group per 1,000 women in that same group. For each reporting area, abortions for women of unknown race were distributed according to the distribution of abortions among women of known race for that area.

*** Number of abortions obtained by women in a given racial group per 1,000 live births to women in that same racial group. For each reporting area, abortions for women of unknown race were distributed according to the distribution of abortions among women of known race for that area.

**TABLE 14:** Reported abortions, by known ethnicity of women who obtained an abortion and reporting area of occurrence — selected reporting areas,* United States, 2014

State/Area	Ethnicity		Total abortions reported by known ethnicity
	Hispanic	Non-Hispanic	
	No. (%)^†^	No. (%)	No. (% of all reported abortions)^§^
Alabama	331 (4.1)	7,718 (95.9)	**8,049 (99.6)**
Alaska	33 (2.5)	1,279 (97.5)	**1,312 (86.4)**
Arizona	4,562 (35.4)	8,338 (64.6)	**12,900 (100.0)**
Arkansas	226 (5.3)	4,022 (94.7)	**4,248 (99.9)**
Colorado	2,442 (25.5)	7,139 (74.5)	**9,581 (90.0)**
Delaware	355 (12.1)	2,581 (87.9)	**2,936 (100.0)**
District of Columbia^¶^	315 (11.3)	2,465 (88.7)	**2,780 (99.6)**
Georgia	1,323 (4.8)	26,373 (95.2)	**27,696 (92.3)**
Hawaii	226 (11.1)	1,804 (88.9)	**2,030 (94.6)**
Idaho	209 (15.9)	1,107 (84.1)	**1,316 (97.3)**
Indiana	601 (7.5)	7,406 (92.5)	**8,007 (98.6)**
Kansas	845 (11.8)	6,329 (88.2)	**7,174 (99.4)**
Michigan	1,018 (3.7)	26,540 (96.3)	**27,558 (99.7)**
Minnesota	606 (6.5)	8,751 (93.5)	**9,357 (92.4)**
Mississippi	55 (2.8)	1,935 (97.2)	**1,990 (86.4)**
Missouri	139 (2.8)	4,887 (97.2)	**5,026 (99.3)**
Montana	79 (4.7)	1,611 (95.3)	**1,690 (100.0)**
Nevada	2,201 (29.8)	5,177 (70.2)	**7,378 (90.7)**
New Jersey**	4,415 (19.3)	18,425 (80.7)	**22,840 (94.5)**
New Mexico	2,124 (55.4)	1,713 (44.6)	**3,837 (85.3)**
New York	24,588 (25.4)	72,123 (74.6)	**96,711 (100.0)**
New York City^††^	20,371 (30.1)	47,249 (69.9)	**67,620 (100.0)**
New York State	4,217 (14.5)	24,874 (85.5)	**29,091 (100.0)**
North Carolina	2,363 (10.4)	20,407 (89.6)	**22,770 (92.5)**
Ohio	794 (4.1)	18,528 (95.9)	**19,322 (91.2)**
Oregon	992 (12.4)	6,988 (87.6)	**7,980 (97.0)**
Pennsylvania	2,845 (8.9)	29,015 (91.1)	**31,860 (99.2)**
South Carolina	238 (4.2)	5,471 (95.8)	**5,709 (99.9)**
South Dakota	36 (6.5)	515 (93.5)	**551 (100.0)**
Tennessee	513 (4.2)	11,616 (95.8)	**12,129 (98.0)**
Texas^§§^	20,004 (37.1)	33,889 (62.9)	**53,893 (99.5)**
Utah	542 (18.9)	2,325 (81.1)	**2,867 (97.3)**
Vermont	30 (2.5)	1,186 (97.5)	**1,216 (98.5)**
Virginia	2,061 (10.3)	17,937 (89.7)	**19,998 (99.1)**
Washington	2,022 (13.4)	13,102 (86.6)	**15,124 (85.4)**
West Virginia	9 (0.5)	1,721 (99.5)	**1,730 (100.0)**
Wisconsin^¶¶^	595 (10.5)	5,045 (89.5)	**5,640 (100.0)**
**Total**	**79,737 (17.1)**	**385,468 (82.9)**	**465,205** (96.8)***
**Abortion rate^†††^**	**11.9**	**11.6**	**11.6**
**Abortion ratio^§§§^**	**156**	**190**	**183**

* Data from 36 reporting areas; excludes 16 areas (California, Connecticut, Florida, Illinois, Iowa, Kentucky, Louisiana, Maine, Maryland, Massachusetts, Nebraska, New Hampshire, North Dakota, Oklahoma, Rhode Island, and Wyoming) that did not report, did not report by ethnicity, or did not meet reporting standards.

^†^ Percentages for the individual component categories might not add to 100 because of rounding.

^§^ Percentage is calculated as the number of abortions reported by known ethnicity divided by the sum of abortions reported by known and unknown ethnicity.

^¶^ Because reporting is not mandatory, a complete number of abortions performed in the District of Columbia could not be obtained.

** Data from hospitals and licensed ambulatory care facilities only; because reporting is not mandatory for private physicians and women’s centers, information could not be obtained for all abortions performed in New Jersey.

^††^ Non-Hispanic category includes abortions for women whose ethnicity was reported as unknown; previous evaluation has shown that most reports without ethnicity are for non-Hispanic women.

^§§^ Reporting form contains only one question for race and ethnicity; therefore, abortions reported for women of white, black, and other races (Asian and Native American) are not explicitly identified as non-Hispanic.

^¶¶^ Includes residents only.

*** Percentage is based on 480,689 abortions reported among the areas that met reporting standards for ethnicity.

^†††^ Number of abortions obtained by women in a given ethnic group per 1,000 women in that same group. For each reporting area, abortions for women of unknown ethnicity were distributed according to the distribution of abortions among women of known ethnicity for that area.

^§§§^ Number of abortions obtained by women in a given ethnic group per 1,000 live births to women in that same ethnic group. For each reporting area, abortions for women of unknown ethnicity were distributed according to the distribution of abortions among women of known ethnicity for that area.

Among the 20 areas^¶¶¶^ that reported by race/ethnicity for 2007 (the first year with sufficient data), 2009, 2010, and 2014, abortion rates decreased substantially for all three major race/ethnicity groups: for non-Hispanic white women, the abortion rate decreased 26% (from 9.4 abortions per 1,000 women in 2007 to 7.0 in 2014), for non-Hispanic black women it decreased 27% (from 36.5 abortions per 1,000 women in 2007 to 26.7 in 2014), and for Hispanic women it decreased 41% (from 20.8 abortions per 1,000 women in 2007 to 12.3 in 2014). For women in all three major race/ethnicity groups, abortion rates decreased from 2007 to 2009 and from 2010 to 2014, but the decreases were greater during the later period, especially for non-Hispanic black and Hispanic women. From 2007 to 2009, the abortion rates decreased 6% for non-Hispanic white women (from 9.4 to 8.8 abortions per 1,000), 1% for non-Hispanic black women (from 36.5 to 36.3 abortions per 1,000), and 8% for Hispanic women (from 20.8 to 19.1 abortions per 1,000); by contrast from 2010 to 2014, the abortion rates decreased 18% for non-Hispanic white women (from 8.5 to 7.0 abortions per 1,000), 23% for non-Hispanic black women (from 34.9 to 26.7 abortions per 1,000), and 35% for Hispanic women (from 19.0 to 12.3 abortions per 1,000).

Abortion ratios also decreased from 2007 to 2014 for all three major race/ethnicity groups: for non-Hispanic white women, the abortion ratio decreased 23% (from 147 abortions per 1,000 live births in 2007 to 113 in 2014), for non-Hispanic black women it decreased 19% (from 514 abortions per 1,000 live births in 2007 to 417 in 2014), and for Hispanic women it decreased 22% (from 205 abortions per 1,000 live births in 2007 to 160 in 2014). As with abortion rates, decreases in abortion ratios were greater for women in all three major race/ethnicity groups from 2010 to 2014 as compared with 2007 to 2009, with the difference in the change between the two periods being most notable for non-Hispanic black and Hispanic women. From 2007 to 2009, abortion ratios decreased 3% among non-Hispanic white women (from 147 to 142 abortions per 1,000 live births) but increased 4% among non-Hispanic black women (from 514 to 535 abortions per 1,000 live births) and did not change among Hispanic women (205 abortions per 1,000 live births). By contrast, from 2010 to 2014, abortion ratios decreased among women in all three race/ethnicity groups. The abortion ratio decreased 18% for non-Hispanic white women (from 138 to 113 abortions per 1,000 live births), 21% for non-Hispanic black women (from 531 to 417 abortions per 1,000 live births), and 28% for Hispanic women (from 222 to 160 abortions per 1,000 live births).

### Marital Status

Among the 42 areas that reported by marital status for 2014, 14.5% of all women who obtained an abortion were married, and 85.5% were unmarried (Table 15). The abortion ratio was 43 abortions per 1,000 live births for married women and 373 abortions per 1,000 live births for unmarried women. Among the 35 reporting areas**** that provided these data for the relevant years of comparison (2005 versus 2014, 2005 versus 2009, 2010 versus 2014, and 2013 versus 2014), the percentage of abortions among unmarried women increased 3% from 2005 to 2014 (from 83.5% to 85.7%), with a larger increase from 2005 to 2009 (2%) than from 2010 to 2014 (<1%). Among unmarried women, the abortion ratio decreased 23% from 2005 to 2014 (from 481 to 369 abortions per 1,000 live births), with a larger decrease also occurring from 2010 to 2014 (16%) than from 2005 to 2009 (10%). Among married women, the abortion ratio decreased 25% from 2005 to 2014 (from 55 to 41 abortions per 1,000 live births), with a larger decrease occurring from 2010 to 2014 (18%) than from 2005 to 2009 (7%).

**TABLE 15:** Reported abortions, by known marital status and reporting area of occurrence — selected reporting areas,* United States, 2014

State/Area	Marital status		Total abortions reported by known marital status
	Married	Unmarried	
	No. (%)^†^	No. (%)	No. (% of all reported abortions)^§^
Alabama	921 (11.4)	7,144 (88.6)	**8,065 (99.8)**
Alaska	250 (18.6)	1,091 (81.4)	**1,341 (88.3)**
Arizona	1,283 (9.9)	11,617 (90.1)	**12,900 (100.0)**
Arkansas	562 (13.2)	3,680 (86.8)	**4,242 (99.7)**
Colorado	1,823 (17.9)	8,379 (82.1)	**10,202 (95.8)**
Delaware	339 (11.5)	2,598 (88.5)	**2,937 (100.0)**
Georgia	4,329 (15.1)	24,250 (84.9)	**28,579 (95.2)**
Hawaii	520 (24.9)	1,572 (75.1)	**2,092 (97.4)**
Idaho	223 (16.8)	1,103 (83.2)	**1,326 (98.0)**
Illinois	3,783 (13.0)	25,239 (87.0)	**29,022 (87.3)**
Indiana	1,087 (13.4)	6,999 (86.6)	**8,086 (99.6)**
Iowa	614 (15.3)	3,406 (84.7)	**4,020** (**100.0)**
Kansas	1,120 (15.6)	6,062 (84.4)	**7,182 (99.5)**
Kentucky	474 (13.8)	2,968 (86.2)	**3,442 (100.0)**
Louisiana	1,179 (11.9)	8,725 (88.1)	**9,904 (96.0)**
Maine	313 (15.9)	1,656 (84.1)	**1,969 (97.4)**
Massachusetts	2,434 (14.8)	14,032 (85.2)	**16,466 (85.1)**
Michigan	2,815 (10.2)	24,802 (89.8)	**27,617 (100.0)**
Minnesota	1,423 (15.3)	7,880 (84.7)	**9,303 (91.9)**
Mississippi	184 (8.4)	1,998 (91.6)	**2,182 (94.7)**
Missouri	760 (15.3)	4,212 (84.7)	**4,972 (98.3)**
Montana	272 (16.3)	1,398 (83.7)	**1,670 (98.8)**
Nebraska	331 (15.2)	1,841 (84.8)	**2,172 (95.7)**
Nevada	1,852 (23.1)	6,155 (76.9)	**8,007 (98.5)**
New Jersey^¶^	2,773 (12.0)	20,290 (88.0)	**23,063 (95.4)**
New Mexico	606 (14.1)	3,680 (85.9)	**4,286 (95.2)**
New York City	8,908 (15.1)	50,201 (84.9)	**59,109 (87.4)**
North Dakota	175 (13.9)	1,084 (86.1)	**1,259 (99.6)**
Ohio	2,703 (14.6)	15,758 (85.4)	**18,461 (87.1)**
Oklahoma	873 (17.8)	4,042 (82.2)	**4,915** (**100.0)**
Oregon	1,291 (18.3)	5,752 (81.7)	**7,043 (85.6)**
Pennsylvania	3,822 (11.9)	28,293 (88.1)	**32,115** (**100.0)**
Rhode Island	527 (20.3)	2,068 (79.7)	**2,595 (86.8)**
South Carolina	475 (8.3)	5,235 (91.7)	**5,710 (99.9)**
South Dakota	92 (16.7)	459 (83.3)	**551 (100.0)**
Tennessee	1,495 (12.5)	10,442 (87.5)	**11,937 (96.5)**
Texas	9,119 (16.9)	44,987 (83.1)	**54,106 (99.9)**
Utah	751 (26.7)	2,065 (73.3)	**2,816 (95.5)**
Vermont	172 (14.4)	1,024 (85.6)	**1,196 (96.8)**
Virginia	3,234 (16.7)	16,142 (83.3)	**19,376 (96.0)**
West Virginia	340 (19.7)	1,390 (80.3)	**1,730 (100.0)**
Wisconsin	867 (15.0)	4,922 (85.0)	**5,789 (99.8)**
**Total**	**67,114 (14.5)**	**396,641 (85.5)**	463,755 (94.5)**
**Abortion ratio^††^**	**43**	**373**	**176**

* Data from 42 reporting areas; excludes 10 areas (California, Connecticut, District of Columbia, Florida, Maryland, New Hampshire, New York State, North Carolina, Washington, and Wyoming) that did not report, did not report by marital status, or did not meet reporting standards.

^†^ Percentages for the individual component categories might not add to 100 because of rounding.

^§^ Percentage is calculated as the number of abortions reported by known marital status divided by the sum of abortions reported by known and unknown marital status.

^¶^ Data from hospitals and licensed ambulatory care facilities only; because reporting is not mandatory for private physicians and women’s centers, information could not be obtained for all abortions performed in New Jersey.

** Percentage is based on 490,499 abortions reported among the areas that met reporting standards for marital status.

^††^ Number of abortions obtained by women by marital status per 1,000 live births to women of the same marital status. For each reporting area, abortions for women of unknown marital status were distributed according to the distribution of abortions among women of known marital status for that area.

### Previous Live Births and Abortions

Data from the 40 areas that reported the number of previous live births for women who obtained abortions in 2014 indicate that 40.4%, 45.7%, and 13.8% of these women had zero, one to two, or three or more previous live births, respectively (Table 16). Among the 32 reporting areas^††††^ that provided these data for the relevant years of comparison (2005 versus 2014, 2005 versus 2009, 2010 versus 2014, and 2013 versus 2014), the percentage of women obtaining abortions who had no previous live births was stable; by contrast, the percentage decreased for women who had one to two previous live births and increased for women who had three or more previous live births. Among the areas included in this comparison, 40.4%, 47.0%, and 12.6% of women had zero, one to two, or three or more previous live births, respectively, in 2005; 40.8%, 45.5%, and 13.7% of women had zero, one to two, or three or more previous live births, respectively, in 2014.

**TABLE 16:** Reported abortions, by known number of previous live births and reporting area of occurrence — selected reporting areas,* United States, 2014

State/Area	No. of previous live births					Total reported by known number of prior live births
	0	1	2	3	≥4	
	No. (%)^†^	No. (%)	No. (%)	No. (%)	No. (%)	No. (% of all reported abortions)^§^
Alabama	2,893 (35.8)	2,399 (29.7)	1,706 (21.1)	680 (8.4)	397 (4.9)	**8,075 (99.9)**
Alaska	667 (44.6)	350 (23.4)	266 (17.8)	120 (8.0)	93 (6.2)	**1,496 (98.6)**
Arizona	5,292 (41.7)	3,009 (23.7)	2,463 (19.4)	1,135 (8.9)	790 (6.2)	**12,689 (98.4)**
Arkansas	1,424 (33.5)	1,222 (28.7)	923 (21.7)	464 (10.9)	218 (5.1)	**4,251 (100.0)**
Colorado	5,528 (52.1)	2,193 (20.7)	1,698 (16.0)	768 (7.2)	418 (3.9)	**10,605 (99.6)**
Delaware	1,149 (39.4)	767 (26.3)	566 (19.4)	275 (9.4)	161 (5.5)	**2,918 (99.4)**
Georgia	10,885 (40.1)	6,885 (25.4)	5,304 (19.6)	2,456 (9.1)	1,593 (5.9)	**27,123 (90.4)**
Hawaii	961 (49.6)	393 (20.3)	327 (16.9)	155 (8.0)	103 (5.3)	**1,939 (90.3)**
Idaho	636 (47.0)	304 (22.5)	228 (16.9)	117 (8.7)	67 (5.0)	**1,352 (99.9)**
Indiana	3,037 (37.6)	2,135 (26.4)	1,688 (20.9)	787 (9.7)	434 (5.4)	**8,081 (99.5)**
Iowa	1,727 (43.0)	880 (21.9)	791 (19.7)	382 (9.5)	238 (5.9)	**4,018 (100.0)**
Kansas	2,912 (40.3)	1,739 (24.1)	1,486 (20.6)	706 (9.8)	376 (5.2)	**7,219 (100.0)**
Kentucky	1,325 (38.5)	943 (27.4)	696 (20.2)	298 (8.7)	180 (5.2)	**3,442 (100.0)**
Louisiana	3,265 (31.6)	3,000 (29.1)	2,391 (23.2)	1,029 (10.0)	631 (6.1)	**10,316 (99.9)**
Maine	1,024 (50.7)	493 (24.4)	329 (16.3)	—^¶^	—	**2,021 (100.0)**
Michigan**	10,143 (37.5)	7,651 (28.3)	5,713 (21.1)	2,524 (9.3)	1,006 (3.7)	**27,037 (97.9)**
Minnesota	4,186 (41.4)	2,401 (23.7)	1,942 (19.2)	954 (9.4)	632 (6.2)	**10,115 (99.9)**
Mississippi	707 (30.7)	714 (31.0)	541 (23.5)	232 (10.1)	109 (4.7)	**2,303 (100.0)**
Missouri	1,753 (34.6)	1,432 (28.3)	1,016 (20.1)	509 (10.1)	350 (6.9)	**5,060 (100.0)**
Montana	847 (50.1)	414 (24.5)	260 (15.4)	110 (6.5)	59 (3.5)	**1,690 (100.0)**
Nebraska	904 (39.8)	541 (23.8)	440 (19.4)	233 (10.3)	152 (6.7)	**2,270 (100.0)**
Nevada	3,183 (39.6)	2,028 (25.2)	1,621 (20.2)	692 (8.6)	515 (6.4)	**8,039 (98.9)**
New Jersey^††^	10,218 (42.5)	6,497 (27.0)	4,330 (18.0)	1,858 (7.7)	1,165 (4.8)	**24,068 (99.5)**
New Mexico	1,503 (37.6)	1,085 (27.2)	764 (19.1)	409 (10.2)	234 (5.9)	**3,995 (88.8)**
New York City	29,073 (44.5)	16,854 (25.8)	11,892 (18.2)	4,742 (7.3)	2,738 (4.2)	**65,299 (96.6)**
North Dakota	515 (40.7)	290 (22.9)	248 (19.6)	133 (10.5)	78 (6.2)	**1,264 (100.0)**
Ohio	7,464 (36.1)	5,676 (27.4)	4,377 (21.1)	1,989 (9.6)	1,196 (5.8)	**20,702 (97.7)**
Oklahoma	2,008 (40.9)	1,222 (24.9)	1,036 (21.1)	430 (8.8)	218 (4.4)	**4,914 (100.0)**
Oregon	3,780 (48.3)	1,730 (22.1)	1,409 (18.0)	580 (7.4)	335 (4.3)	**7,834 (95.2)**
Pennsylvania	12,448 (38.7)	8,873 (27.6)	6,360 (19.8)	2,787 (8.7)	1,658 (5.2)	**32,126 (100.0)**
Rhode Island	1,329 (44.9)	775 (26.2)	531 (18.0)	206 (7.0)	116 (3.9)	**2,957 (98.9)**
South Carolina	2,382 (41.7)	1,525 (26.7)	1,153 (20.2)	431 (7.5)	223 (3.9)	**5,714 (100.0)**
South Dakota	197 (35.8)	135 (24.5)	130 (23.6)	52 (9.4)	37 (6.7)	**551 (100.0)**
Tennessee	4,242 (34.9)	3,340 (27.5)	2,590 (21.3)	1,150 (9.5)	817 (6.7)	**12,139 (98.1)**
Texas	20,171 (37.3)	14,003 (25.9)	11,484 (21.2)	5,440 (10.0)	3,047 (5.6)	**54,145 (100.0)**
Utah	1,407 (48.0)	633 (21.6)	499 (17.0)	228 (7.8)	163 (5.6)	**2,930 (99.4)**
Vermont	666 (54.2)	272 (22.1)	186 (15.1)	68 (5.5)	37 (3.0)	**1,229 (99.5)**
Virginia	7,910 (39.3)	5,402 (26.8)	4,140 (20.6)	1,764 (8.8)	926 (4.6)	**20,142 (99.8)**
Washington	8,151 (46.0)	4,188 (23.7)	3,231 (18.2)	1,356 (7.7)	781 (4.4)	**17,707 (100.0)**
West Virginia	550 (31.8)	530 (30.6)	404 (23.4)	168 (9.7)	78 (4.5)	**1,730 (100.0)**
**Total**	**178,462 (40.4)**	**114,923 (26.0)**	**87,159 (19.7)**	**38,588 (8.7)**	**22,373 (5.1)**	**441,505** **(98.2)**^§§^

* Data from 40 reporting areas; excludes 12 areas (California, Connecticut, District of Columbia, Florida, Illinois, Maryland, Massachusetts, New Hampshire, New York State, North Carolina, Wisconsin, and Wyoming) that did not report, did not report by number of previous births, or did not meet reporting standards.

^†^ Percentages for the individual component categories might not add to 100 because of rounding.

^§^ Percentage is calculated as the number of abortions reported by known number of previous live births, divided by the sum of abortions reported by known and unknown number of previous live births.

^¶^ Cell details are not displayed because of small numbers (n = 1–4).

** Recorded as the number of previous pregnancies carried to term.

^††^ Data from hospitals and licensed ambulatory care facilities only; because reporting is not mandatory for private physicians and women’s centers, information could not be obtained for all abortions performed in New Jersey.

^§§ ^Percentage is based on 449,808 abortions reported among the areas that met reporting standards for the number of previous births.

Data from the 40 areas that reported the number of previous abortions for women who obtained abortions in 2014 indicate that the majority (55.1%) had no previous abortions, 36.3% had one to two previous abortions, and 8.6% had three or more previous abortions (Table 17). Among the 33 reporting areas^§§§§^ that provided data for the relevant years of comparison (2005 to 2014, 2005 versus 2009, 2010 versus 2014, and 2013 versus 2014), the percentage of women who had zero or one to two previous abortions did not change substantially over time, but the percentage of women who had three or more previous abortions increased from 2005 to 2014. Among the areas included in this comparison, 55.5%, 36.6%, and 7.9% of women had zero, one to two, or three or more previous abortions, respectively, in 2005; 55.3%, 36.1%, and 8.6% of women had zero, one to two, or three or more previous abortions, respectively, in 2014.

**TABLE 17:** Reported abortions, by known number of previous induced abortions and reporting area of occurrence — selected reporting areas,* United States, 2014

State/Area	Number of previous induced abortions				Total abortions reported by known no. of previous induced abortions
	0	1	2	≥3	
	No. (%)^†^	No. (%)	No. (%)	No. (%)	No. (% of all reported abortions)^§^
Alabama	5,181 (64.2)	1,909 (23.6)	685 (8.5)	300 (3.7)	**8,075 (99.9)**
Alaska	1,050 (69.5)	288 (19.1)	114 (7.5)	58 (3.8)	**1,510 (99.5)**
Arizona	7,985 (63.2)	3,249 (25.7)	966 (7.6)	439 (3.5)	**12,639 (98.0)**
Arkansas	2,457 (57.8)	979 (23.0)	461 (10.8)	354 (8.3)	**4,251 (100.0)**
Colorado	6,999 (66.0)	2,617 (24.7)	702 (6.6)	290 (2.7)	**10,608 (99.6)**
Delaware	1,588 (54.6)	803 (27.6)	321 (11.0)	199 (6.8)	**2,911 (99.1)**
Georgia	17,882 (66.7)	5,324 (19.9)	2,314 (8.6)	1,291 (4.8)	**26,811 (89.3)**
Hawaii	1,129 (55.8)	525 (26.0)	229 (11.3)	139 (6.9)	**2,022 (94.2)**
Idaho	937 (69.3)	292 (21.6)	87 (6.4)	36 (2.7)	**1,352 (99.9)**
Indiana	5,173 (63.9)	1,986 (24.5)	640 (7.9)	301 (3.7)	**8,100 (99.8)**
Iowa	2,583 (64.3)	980 (24.4)	309 (7.7)	144 (3.6)	**4,016 (99.9)**
Kansas	4,671 (64.7)	1,748 (24.2)	517 (7.2)	283 (3.9)	**7,219 (100.0)**
Kentucky	2,153 (62.6)	801 (23.3)	294 (8.5)	194 (5.6)	**3,442 (100.0)**
Louisiana	6,241 (60.5)	2,690 (26.1)	984 (9.5)	402 (3.9)	**10,317 (100.0)**
Maine	1,315 (65.1)	454 (22.5)	171 (8.5)	80 (4.0)	**2,020 (100.0)**
Massachusetts	9,651 (52.7)	4,738 (25.9)	2,270 (12.4)	1,654 (9.0)	**18,313 (94.6)**
Michigan	13,693 (49.6)	7,198 (26.1)	3,885 (14.1)	2,853 (10.3)	**27,629 (100.0)**
Minnesota	6,049 (59.8)	2,411 (23.8)	978 (9.7)	680 (6.7)	**10,118 (100.0)**
Mississippi	1,519 (66.0)	506 (22.0)	204 (8.9)	74 (3.2)	**2,303 (100.0)**
Missouri	3,076 (60.8)	1,342 (26.5)	435 (8.6)	206 (4.1)	**5,059 (100.0)**
Montana	427 (25.3)	934 (55.3)	220 (13.0)	109 (6.4)	**1,690 (100.0)**
Nebraska	1,460 (64.3)	564 (24.8)	168 (7.4)	78 (3.4)	**2,270 (100.0)**
Nevada	3,833 (48.0)	2,477 (31.0)	1,016 (12.7)	660 (8.3)	**7,986 (98.2)**
New Jersey^¶^	15,839 (65.8)	4,357 (18.1)	2,070 (8.6)	1,802 (7.5)	**24,068 (99.5)**
New York City	26,371 (40.9)	15,244 (23.7)	10,956 (17.0)	11,855 (18.4)	**64,426 (95.3)**
North Dakota	843 (66.7)	266 (21.0)	99 (7.8)	56 (4.4)	**1,264 (100.0)**
Ohio	7,464 (36.1)	5,676 (27.4)	4,377 (21.1)	3,185 (15.4)	**20,702 (97.7)**
Oklahoma	3,327 (67.7)	1,050 (21.4)	338 (6.9)	196 (4.0)	**4,911 (99.9)**
Oregon	4,727 (59.3)	1,911 (24.0)	776 (9.7)	560 (7.0)	**7,974 (96.9)**
Pennsylvania	17,203 (53.5)	8,024 (25.0)	3,904 (12.2)	2,995 (9.3)	**32,126 (100.0)**
Rhode Island	1,530 (52.5)	783 (26.9)	352 (12.1)	248 (8.5)	**2,913 (97.4)**
South Carolina	3,203 (56.1)	1,427 (25.0)	618 (10.8)	466 (8.2)	**5,714 (100.0)**
South Dakota	365 (66.2)	126 (22.9)	36 (6.5)	24 (4.4)	**551 (100.0)**
Tennessee	6,429 (52.9)	3,253 (26.7)	1,432 (11.8)	1,049 (8.6)	**12,163 (98.3)**
Texas	31,578 (58.3)	14,436 (26.7)	5,314 (9.8)	2,802 (5.2)	**54,130 (100.0)**
Utah	2,183 (74.1)	551 (18.7)	135 (4.6)	79 (2.7)	**2,948 (100.0)**
Vermont	800 (65.1)	258 (21.0)	95 (7.7)	76 (6.2)	**1,229 (99.5)**
Virginia	11,130 (55.2)	5,395 (26.8)	2,292 (11.4)	1,350 (6.7)	**20,167 (99.9)**
Washington	10,142 (57.3)	4,271 (24.1)	1,876 (10.6)	1,417 (8.0)	**17,706 (100.0)**
West Virginia	922 (53.3)	494 (28.6)	187 (10.8)	127 (7.3)	**1,730 (100.0)**
**Total**	**251,108 (55.1)**	**112,337 (24.7)**	**52,827 (11.6)**	**39,111 (8.6)**	455,383 (98.0)**

* Data from 40 reporting areas; excludes 12 areas (California, Connecticut, District of Columbia, Florida, Illinois, Maryland, New Hampshire, New Mexico, New York State, North Carolina, Wisconsin, and Wyoming) that did not report, did not report by the number of previous induced abortions, or did not meet reporting standards.

^†^ Percentages for the individual component categories might not add to 100 because of rounding.

^§^ Percentage is calculated as the number of abortions reported by known number of previous induced abortions divided by the sum of abortions reported by known and unknown number of previous induced abortions.

^¶^ Data from hospitals and licensed ambulatory care facilities only; because reporting is not mandatory for private physicians and women’s centers, information could not be obtained for all abortions performed in New Jersey.

** Percentage is based on 464,662 abortions reported among the areas that met reporting standards for the number of previous abortions.

### Maternal Age and Marital Status by Race/Ethnicity

In certain reporting areas, abortions that were categorized by maternal race and race/ethnicity were further categorized by maternal age and by marital status (Tables 18 and 19). A consistent pattern existed for abortions by maternal age across all race/ethnicity groups, with the smallest percentage of abortions occurring among adolescents aged <15 years (0.2%–0.4%) and the largest percentage occurring among women aged 20–24 years (27.3%–33.8%) (Table 19). A consistent pattern also existed for abortions by marital status across all race/ethnicity groups, with a higher percentage of abortions occurring among women who were unmarried (68.5%–91.9%) than among those who were married (8.1%–31.5%) (Table 19). However, for abortions among unmarried women, the percentage was higher for non-Hispanic black women (91.9%) than for non-Hispanic white (83.1%) or Hispanic women (84.7%) (Table 19).

**TABLE 18:** Reported abortions, by known race, age group, and marital status of women who obtained an abortion — selected reporting areas, United States, 2014

Characteristic	Race			Total
	White	Black	Other	
	No. (%)*	No. (%)	No. (%)	No. (%)
**Age group (yrs)^†^**				
<15	354 (0.3)	408 (0.4)	69 (0.2)	**831 (0.3)**
15–19	14,802 (10.6)	10,848 (10.0)	2,599 (9.3)	**28,249 (10.2)**
15	721 (0.5)	695 (0.6)	132 (0.5)	**1,548 (0.6)**
16	1,386 (1.0)	1,155 (1.1)	231 (0.8)	**2,772 (1.0)**
17	2,226 (1.6)	1,671 (1.5)	391 (1.4)	**4,288 (1.6)**
18	4,463 (3.2)	2,971 (2.8)	746 (2.7)	**8,180 (3.0)**
19	6,006 (4.3)	4,356 (4.0)	1,099 (3.9)	**11,461 (4.2)**
20–24	45,405 (32.4)	37,028 (34.3)	8,008 (28.7)	**90,441 (32.8)**
25–29	36,610 (26.1)	30,106 (27.9)	7,137 (25.6)	**73,853 (26.8)**
30–34	23,541 (16.8)	17,996 (16.7)	5,119 (18.3)	**46,656 (16.9)**
35–39	13,802 (9.9)	8,963 (8.3)	3,443 (12.3)	**26,208 (9.5)**
≥40	5,582 (4.0)	2,623 (2.4)	1,548 (5.5)	**9,753 (3.5)**
**Total**	**140,096 (100.0)**	**107,972 (100.0)**	**27,923 (100.0)**	**275,991 (100.0)**
**Marital status^§^**				
Married	19,366 (15.8)	6,827 (7.5)	6,648 (26.1)	**32,841 (13.7)**
Unmarried	103,068 (84.2)	84,383 (92.5)	18,785 (73.9)	**206,236 (86.3)**
**Total**	**122,434 (100.0)**	**91,210 (100.0)**	**25,433 (100.0)**	**239,077 (100.0)**

* Percentages for the individual component categories might not add to 100 because of rounding.

^†^ Data from 35 reporting areas; excludes 17 areas (California, Connecticut, Florida, Illinois, Kentucky, Maryland, Massachusetts, Nevada, New Hampshire, New Mexico, New York State, New York City, Pennsylvania, Texas, Utah, Washington, and Wyoming) that did not report, did not report by race or age, or did not meet reporting standards.

^§^ Data from 32 reporting areas; excludes 20 areas (California, Connecticut, District of Columbia, Florida, Illinois, Kentucky, Maryland, Massachusetts, Nevada, New Hampshire, New Mexico, New York State, New York City, North Carolina, Pennsylvania, Texas, Utah, Washington, Wisconsin, and Wyoming) that did not report, did not report by race or marital status, or did not meet reporting standards.

**TABLE 19:** Reported abortions, by known race/ethnicity, age group, and marital status of women who obtained an abortion — selected reporting areas, United States, 2014

Characteristic	Non-Hispanic			Hispanic	Total
	White	Black	Other		
	No. (%)*	No. (%)	No. (%)	No. (%)	No. (%)
**Age group (yrs)^†^**					
<15	297 (0.2)	470 (0.4)	42 (0.2)	160 (0.3)	**969 (0.3)**
15–19	13,485 (10.2)	13,396 (10.6)	2,129 (8.0)	5,847 (11.3)	**34,857 (10.3)**
15	647 (0.5)	828 (0.7)	99 (0.4)	293 (0.6)	**1,867 (0.6)**
16	1,228 (0.9)	1,443 (1.1)	181 (0.7)	552 (1.1)	**3,404 (1.0)**
17	2,101 (1.6)	2,135 (1.7)	330 (1.2)	959 (1.9)	**5,525 (1.6)**
18	4,042 (3.0)	3,722 (2.9)	591 (2.2)	1,693 (3.3)	**10,048 (3.0)**
19	5,467 (4.1)	5,268 (4.2)	928 (3.5)	2,350 (4.5)	**14,013 (4.2)**
20–24	42,306 (31.9)	42,752 (33.8)	7,231 (27.3)	16,033 (31.0)	**108,322 (32.1)**
25–29	35,188 (26.5)	34,436 (27.2)	6,864 (25.9)	13,842 (26.7)	**90,330 (26.8)**
30–34	22,597 (17.0)	21,256 (16.8)	5,126 (19.4)	8,983 (17.4)	**57,962 (17.2)**
35–39	13,243 (10.0)	10,933 (8.6)	3,498 (13.2)	5,070 (9.8)	**32,744 (9.7)**
≥40	5,430 (4.1)	3,328 (2.6)	1,596 (6.0)	1,820 (3.5)	**12,174 (3.6)**
**Total**	132,546 (100.0)	126,571 (100.0)	26,486 (100.0)	51,755 (100.0)	**337,358 (100.0)**
**Marital status^§^**					
Married	20,403 (16.9)	9,527 (8.1)	8,610 (31.5)	9,648 (15.3)	**48,188 (14.7)**
Unmarried	100,610 (83.1)	107,593 (91.9)	18,743 (68.5)	53,615 (84.7)	**280,561 (85.3)**
**Total**	**121,013 (100.0)**	**117,120 (100.0)**	**27,353 (100.0)**	**63,263 (100.0)**	**328,749 (100.0)**

* Percentages for the individual component categories might not add to 100 because of rounding.

^†^ Data from 29 reporting areas; excludes 23 areas (Alaska, California, Connecticut, Florida, Illinois, Iowa, Kentucky, Louisiana, Maine, Maryland, Massachusetts, Mississippi, Nebraska, New Hampshire, New Mexico, North Dakota, Oklahoma, Pennsylvania, Rhode Island, Texas, Washington, Wisconsin, and Wyoming) that did not report, did not report by race/ethnicity or age, or did not meet reporting standards.

^§^ Data from 27 reporting areas; excludes 25 reporting areas (Alaska, California, Connecticut, District of Columbia, Florida, Illinois, Iowa, Kentucky, Louisiana, Maine, Maryland, Massachusetts, Mississippi, Nebraska, New Hampshire, New Mexico, New York State, North Carolina, North Dakota, Oklahoma, Pennsylvania, Rhode Island, Washington, Wisconsin, and Wyoming) that did not report, did not report by race/ethnicity or gestational age, or did not meet reporting standards.

### Weeks of Gestation by Maternal Age, Race/Ethnicity, and Method Type

**FIGURE 3:**
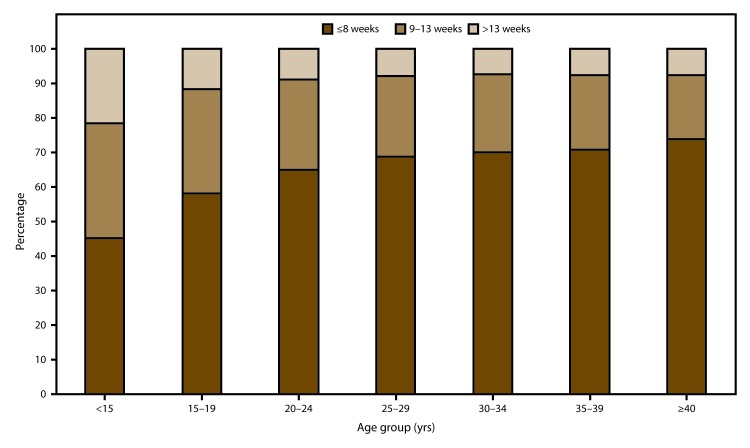
Percentage* distribution of gestational ages at time of abortion, by age of woman — selected reporting areas,^†^ United States, 2014 * Based on the total number of abortions reported with known weeks of gestation. ^†^ Data from 38 reporting areas; excludes 14 reporting areas (California, Connecticut, District of Columbia, Florida, Illinois, Kentucky, Maryland, Massachusetts, New Hampshire, New York State, Pennsylvania, Texas, Wisconsin, and Wyoming) that did not report, did not report by age or gestational age, or did not meet reporting standards.

In certain reporting areas, abortions that were categorized by weeks of gestation were further categorized by maternal age, race, and race/ethnicity (Tables 20 and 21). In every subgroup for these three variables, the largest percentage of abortions occurred at ≤8 weeks’ gestation. However, by maternal age, 43.0% of adolescents aged <15 years and 56.5% of adolescents aged 15–19 years obtained an abortion by ≤8 weeks’ gestation, compared with 63.2%–72.5% of women in older age groups (Figure 3) (Table 20). Conversely, 22.6% of adolescents aged <15 years and 12.5% of adolescents aged 15–19 years obtained an abortion after 13 weeks’ gestation, compared with 8.0%–9.5% for women in older age groups. By race/ethnicity, 58.8% of non-Hispanic black women obtained an abortion at ≤8 weeks’ gestation, compared with 65.7%–70.6% of women from other race/ethnicity groups. Differences in abortions after 13 weeks’ gestation across race/ethnicity groups were less apparent than differences across age groups (10.3% for non-Hispanic black women, compared with 8.1%–8.6% for women in the remaining race/ethnicity groups).

**TABLE 20:** Reported abortions, by known weeks of gestation, age group, and race/ethnicity of women who obtained an abortion — selected reporting areas, United States, 2014

Characteristic	Weeks of gestation					
	≤8	9–13	14–15	16–17	18–20	≥21
	No. (%)	No. (%)	No. (%)	No. (%)	No. (%)	No. (%)
Age group (yrs)*^,†^						
<15	492 (43.0)	394 (34.4)	77 (6.7)	41 (3.6)	57 (5.0)	84 (7.3)
15–19	22,097 (56.5)	12,148 (31.1)	1,751 (4.5)	1,172 (3.0)	1,049 (2.7)	894 (2.3)
20–24	76,653 (63.2)	33,146 (27.3)	4,454 (3.7)	2,827 (2.3)	2,518 (2.1)	1,648 (1.4)
25–29	68,207 (67.1)	24,946 (24.5)	3,325 (3.3)	2,014 (2.0)	1,825 (1.8)	1,333 (1.3)
30–34	44,916 (68.4)	15,531 (23.6)	1,926 (2.9)	1,202 (1.8)	1,238 (1.9)	900 (1.4)
35–39	25,773 (69.5)	8,325 (22.4)	1,157 (3.1)	687 (1.9)	683 (1.8)	468 (1.3)
≥40	10,015 (72.5)	2,691 (19.5)	399 (2.9)	266 (1.9)	278 (2.0)	166 (1.2)
**Total**	**248,153 (65.3)**	**97,181 (25.6)**	**13,089 (3.4)**	**8,209 (2.2)**	**7,648 (2.0)**	**5,493 (1.4)**
Race/Ethnicity*^,§^						
Non-Hispanic						
White	91,531 (67.8)	32,507 (24.1)	4,255 (3.2)	2,580 (1.9)	2,440 (1.8)	1,599 (1.2)
Black	77,919 (58.8)	40,982 (30.9)	5,506 (4.2)	3,421 (2.6)	2,947 (2.2)	1,739 (1.3)
Other	20,621 (70.6)	6,072 (20.8)	894 (3.1)	642 (2.2)	580 (2.0)	380 (1.3)
Hispanic	44,199 (65.7)	17,374 (25.8)	2,439 (3.6)	1,435 (2.1)	1,170 (1.7)	690 (1.0)
**Total**	**234,270 (64.4)**	**96,935 (26.6)**	**13,094 (3.6)**	**8,078 (2.2)**	**7,137 (2.0)**	**4,408 (1.2)**

* Row percentages might not add to 100 because of rounding.

^†^ Data from 38 reporting areas; excludes 14 reporting areas (California, Connecticut, District of Columbia, Florida, Illinois, Kentucky, Maryland, Massachusetts, New Hampshire, New York State, Pennsylvania, Texas, Wisconsin, and Wyoming) that did not report, did not report by age or gestational age, or did not meet reporting standards.

^§^ Data from 28 reporting areas; excludes 24 reporting areas (Alaska, California, Connecticut, District of Columbia, Florida, Illinois, Iowa, Kentucky, Louisiana, Maine, Maryland, Massachusetts, Mississippi, Nebraska, New Hampshire, New Mexico, New York State, North Dakota, Oklahoma, Pennsylvania, Rhode Island, Washington, Wisconsin, and Wyoming) that did not report, did not report by race/ethnicity or gestational age, or did not meet reporting standards.

**TABLE 21:** Reported abortions obtained at ≤13 weeks’ gestation, by known weeks of gestation, age group, and race/ethnicity of women who obtained an abortion — selected reporting areas, United States, 2014

Characteristic	Weeks of gestation							
	≤6	7	8	9	10	11	12	13
	No. (%)	No. (%)	No. (%)	No. (%)	No. (%)	No. (%)	No. (%)	No. (%)
Age group (yrs)*^,†^								
<15	207 (23.4)	161 (18.2)	124 (14.0)	107 (12.1)	86 (9.7)	83 (9.4)	62 (7.0)	56 (6.3)
15–19	10,374 (30.3)	6,278 (18.3)	5,445 (15.9)	3,813 (11.1)	2,827 (8.3)	2,326 (6.8)	1,844 (5.4)	1,338 (3.9)
20–24	38,652 (35.2)	21,282 (19.4)	16,719 (15.2)	10,912 (9.9)	7,640 (7.0)	6,238 (5.7)	4,693 (4.3)	3,663 (3.3)
25–29	36,010 (38.7)	18,453 (19.8)	13,744 (14.8)	8,687 (9.3)	5,652 (6.1)	4,538 (4.9)	3,389 (3.6)	2,680 (2.9)
30–34	23,956 (39.6)	12,059 (19.9)	8,901 (14.7)	5,560 (9.2)	3,503 (5.8)	2,739 (4.5)	2,092 (3.5)	1,637 (2.7)
35–39	13,915 (40.8)	6,887 (20.2)	4,971 (14.6)	2,956 (8.7)	1,795 (5.3)	1,477 (4.3)	1,158 (3.4)	939 (2.8)
≥40	5,739 (45.2)	2,560 (20.1)	1,716 (13.5)	976 (7.7)	569 (4.5)	455 (3.6)	358 (2.8)	333 (2.6)
**Total**	**128,853 (37.3)**	**67,680 (19.6)**	**51,620 (14.9)**	**33,011 (9.6)**	**22,072 (6.4)**	**17,856 (5.2)**	**13,596 (3.9)**	**10,646 (3.1)**
Race/Ethnicity*^,§^								
Non-Hispanic								
White	49,141 (39.6)	24,401 (19.7)	17,989 (14.5)	11,281 (9.1)	7,282 (5.9)	6,090 (4.9)	4,312 (3.5)	3,542 (2.9)
Black	36,151 (30.4)	22,724 (19.1)	19,044 (16.0)	13,473 (11.3)	9,478 (8.0)	7,881 (6.6)	5,838 (4.9)	4,312 (3.6)
Other	11,794 (44.2)	5,245 (19.6)	3,582 (13.4)	2,172 (8.1)	1,350 (5.1)	977 (3.7)	812 (3.0)	761 (2.9)
Hispanic	23,552 (38.3)	11,681 (19.0)	8,966 (14.6)	6,206 (10.1)	4,087 (6.6)	3,142 (5.1)	2,110 (3.4)	1,829 (3.0)
**Total**	**120,638 (36.4)**	**64,051 (19.3)**	**49,581 (15.0)**	**33,132 (10.0)**	**22,197 (6.7)**	**18,090 (5.5)**	**13,072 (3.9)**	**10,444 (3.2)**

* Row percentages might not add to 100 because of rounding.

^†^ Data from 38 reporting areas; excludes 14 reporting areas (California, Connecticut, District of Columbia, Florida, Illinois, Kentucky, Maryland, Massachusetts, New Hampshire, New York State, Pennsylvania, Texas, Wisconsin, and Wyoming) that did not report, did not report by age or gestational age, or did not meet reporting standards.

^§^ Data from 28 reporting areas; excludes 24 reporting areas (Alaska, California, Connecticut, District of Columbia, Florida, Illinois, Iowa, Kentucky, Louisiana, Maine, Maryland, Massachusetts, Mississippi, Nebraska, New Hampshire, New Mexico, New York State, North Dakota, Oklahoma, Pennsylvania, Rhode Island, Washington, Wisconsin, and Wyoming) that did not report, did not report by race or gestational age, or did not meet reporting standards.

Among abortions categorized by weeks of gestation and method type, surgical abortion accounted for the largest percentage of abortions within every gestational age category (Table 22). At ≤8 weeks’ gestation, surgical abortion accounted for a smaller percentage of abortions (66.7%) than at any other stage of gestation. At 9–20 weeks’ gestation, surgical abortion accounted for 96.8%–98.9% of all abortions; this percentage then decreased to 90.1% of abortions at ≥21 weeks’ gestation. By contrast, at ≤8 weeks’ gestation early medical abortion accounted for 33.3% of abortions then decreased to 3.1% at 9–13 weeks and 0.9%–1.2% at 14–17 weeks before increasing to 2.8% at 18–20 weeks and 9.1% at ≥21 weeks. Throughout gestation, abortions performed by intrauterine instillation or hysterectomy/hysterotomy were rare (<0.01%–0.7% of abortions).

**TABLE 22:** Reported abortions, by known weeks of gestation and method type — selected reporting areas,* United States, 2014

Method type	Weeks of gestation						Total
	≤8	9–13	14–15	16–17	18–20	≥21	
	No. (%)^†^	No. (%)	No. (%)	No. (%)	No. (%)	No. (%)	No. (%)
**Surgical^§^**							
≤13 weeks gestation	177,232 (66.7)	103,328 (96.8)	NA	NA	NA	NA	**280,560 (68.5)**
>13 weeks gestation	NA	NA	14,431 (98.9)	9,047 (98.5)	7,848 (96.8)	4,845 (90.1)	**36,171 (8.8)**
**Medical^¶^**							
≤8 weeks gestation	88,604 (33.3)	NA	NA	NA	NA	NA	**88,604 (21.6)**
>8 weeks gestation	NA	3,348 (3.1)	130 (0.9)	112 (1.2)	224 (2.8)	487 (9.1)	**4,301 (1.0)**
**Intrauterine instillation**	—**	18 (0.0)	23 (0.2)	12 (0.1)	28 (0.3)	36 (0.7)	**117 (0.0)**
**Hysterectomy/Hysterotomy**	17 (0.0)	12 (0.0)	4 (0.0)	11 (0.1)	8 (0.1)	8 (0.1)	**60 (0.0)**
**Total**	**265,853 (100.0)**	**106,706 (100.0)**	**14,588 (100.0)**	**9,182 (100.0)**	**8,108 (100.0)**	**5,376 (100.0)**	**409,813 (100.0)**

**Abbreviation:** NA = not applicable.

* Data from 36 reporting areas; excludes 16 areas (California, Connecticut, District of Columbia, Florida, Hawaii, Illinois, Kentucky, Louisiana, Maryland, Massachusetts, New Hampshire, New York State, Pennsylvania, Tennessee, Wisconsin, and Wyoming) that did not report, did not report by method type or gestational age, did not meet reporting standards, or did not have medical abortion as a specific category on their reporting form.

^†^ For each gestational age category, percentages of all method types might not add to 100 because of rounding.

^§^ Includes aspiration curettage, suction curettage, manual vacuum aspiration, menstrual extraction, sharp curettage, and dilation and evacuation procedures.

^¶^ The administration of medication or medications to induce an abortion; at ≤8 weeks’ gestation, typically involves the use of mifepristone and misoprostol; at >8 weeks’ gestation, typically involves the use of vaginal prostaglandins.

** Intrauterine instillations reported at ≤12 weeks’ gestation have not been included with known values.

### Abortion Mortality

Using national data from the Pregnancy Mortality Surveillance System (*49*), CDC identified four abortion-related deaths for 2013 (Table 23). These deaths were identified either by some indication of abortion on the death certificate, by reports from a health care provider or public health agency, or from a media report. Investigation of these cases indicated that all four deaths were related to legal abortion and none to illegal abortion.

**TABLE 23:** Number of deaths and case-fatality rates* for abortion-related deaths reported to CDC, by type of abortion — United States, 1973–2013^†^

Year	Type of abortion				CFR per 100,000 legal abortions
	Induced		Unknown**	Total	
	Legal^§^	Illegal^¶^			
**1973–1977**					**2.09**
1973	25	19	3	47	
1974	26	6	1	33	
1975	29	4	1	34	
1976	11	2	1	14	
1977	17	4	0	21	
**1978–1982**					**0.78**
1978	9	7	0	16	
1979	22	0	0	22	
1980	9	1	2	12	
1981	8	1	0	9	
1982	11	1	0	12	
**1983–1987**					**0.66**
1983	11	1	0	12	
1984	12	0	0	12	
1985	11	1	1	13	
1986	11	0	2	13	
1987	7	2	0	9	
**1988–1992**					**0.74**
1988	16	0	0	16	
1989	12	1	0	13	
1990	9	0	0	9	
1991	11	1	0	12	
1992	10	0	0	10	
**1993–1997**					**0.52**
1993	6	1	2	9	
1994	10	2	0	12	
1995	4	0	0	4	
1996	9	0	0	9	
1997	7	0	0	7	
**1998–2002**					**0.63**
1998	9	0	0	9	
1999	4	0	0	4	
2000	11	0	0	11	
2001	7	1	0	8	
2002	10	0	0	10	
**2003–2007**					**0.60**
2003	10	0	0	10	
2004	7	1	0	8	
2005	7	0	0	7	
2006	7	0	0	7	
2007	6	0	0	6	
**2008–2013**					**0.62**
2008	12	0	0	12	
2009	8	0	0	8	
2010	10	0	0	10	
2011	2	0	0	2	
2012	4	0	0	4	
2013	4	0	0	4	
**Total**	**431**	**56**	**13**	**500**	**0.79**

**Abbreviation:** CFR = case-fatality rate.

* Number of legal induced abortion-related deaths per 100,000 reported legal induced abortions. Because a substantial number of legal induced abortions occurred outside reporting areas that provided data to CDC, national case-fatality rates (i.e., number of legal induced abortion-related deaths per 100,000 reported legal induced abortions in the United States) were calculated with denominator data from a more complete source (*50***,***74*). Case-fatality rates were computed for consecutive 5-year periods during 1973–2007 and then for a consecutive 6-year period during 2008–2013 because rates based on <20 cases are highly variable (*41*).

^†^ Certain numbers might differ from those in reports published previously because additional information has been supplied to CDC subsequent to publication.

^§^ An abortion is defined as legal if it was performed by a licensed clinician within the limits of state law.

^¶^ An abortion is defined as illegal if it was performed by any person other than a licensed clinician.

** Unknown whether abortion was induced or spontaneous.

The annual number of deaths related to legal induced abortion has fluctuated from year to year over the past 40 years (Table 23). For example, nine legal induced abortion-related deaths occurred in 1998, four in 1999, and 11 in 2000. Because of this variability and the relatively small number of legal induced abortion-related deaths every year, national legal abortion case-fatality rates were calculated for consecutive 5-year periods during 1973–2007 and for a consecutive 6-year period during 2008–2013. The national legal induced abortion case-fatality rate for 2008–2013 was 0.62 legal induced abortion-related deaths per 100,000 reported legal abortions. This case-fatality rate was similar to the rate for most of the preceding 5-year periods but lower than the case-fatality rate of 2.09 legal induced abortion-related deaths per 100,000 reported legal abortions for the 5-year period (1973–1977) immediately following nationwide legalization of abortion in 1973. Possible abortion-related deaths that occurred during 2014–2017 are being assessed.

## Discussion

For 2014, a total of 652,639 abortions were reported to CDC. Of these abortions, 642,317 (98.4%) were from 48 reporting areas that submitted data every year during 2005–2014, thus providing the information necessary for evaluating trends. These 48 areas had an abortion rate of 12.1 abortions per 1,000 women aged 15–44 years and an abortion ratio of 193 abortions per 1,000 live births. Among these areas, the number and rate of reported abortions decreased 2%, and the ratio decreased 3%, which, in combination with decreases that occurred during 2010–2013 (*11*–*14*), resulted in the lowest values for the entire period of analysis for all three measures. Among areas that reported by age every year during 2005–2014, women in their 20s accounted for the majority of abortions and had the highest abortion rates, and decreases in the abortion rate for adolescents aged <20 years were greater than the decreases in the abortion rate for any other age group. In addition, throughout the period of analysis, ≤9.1% of abortions were performed after 13 weeks’ gestation; the majority of abortions were performed at ≤8 weeks’ gestation, and this percentage increased from 63.5% in 2005 to 64.7% in 2014. Among areas that included medical abortion on their reporting form every year, the percentage of all abortions performed by early medical abortion increased from 10.7% in 2005 to 22.5% in 2014.

In addition to highlighting changes in abortion that occurred among all women of reproductive age, this report underscores important maternal age differences in abortion trends. During 2005–2014, abortion rates for women in their 20s were consistently higher than for any other age group, and women in their 20s accounted for the majority of abortions (56%–59%); therefore, they have contributed substantially to overall changes. Conversely, during 2005–2014, women aged ≥40 years had consistently low abortion rates and accounted for a small percentage of abortions (≤3.7%); therefore, they have had a much smaller contribution to overall abortion trends. Nonetheless, among women aged ≥40 years, the abortion ratio continues to be higher than among women in their mid to late 20s and 30s. Given the small proportion of abortions that are performed later in gestation among women aged ≥40 years, which potentially might be completed for maternal medical indications or fetal anomalies, the continuing high abortion ratio among these older women suggests that unintended pregnancy is a problem that women encounter throughout their reproductive years (*51*).

The adolescent abortion trends described in this report are important for monitoring progress that has been made toward reducing adolescent pregnancies in the United States. During 1990–2011, the pregnancy rate for adolescents aged 15–19 years decreased 55% to a historic low of 52.4 pregnancies per 1,000; this decrease was associated with a larger decrease in adolescent abortion rates (67%) as compared with birthrates (48%) (*52*). The most recent national birth data indicate that the birthrate for adolescents aged 15–19 years decreased an additional 35% from 2011 to 2016 to an all-time low (*53*,*54*). The 49% decrease from 2005 to 2014 in the abortion rate for adolescents aged 15–19 years as compared with the 40% decrease in the birth rate for this period suggests that adolescent pregnancies in the United States continue to decrease and that this decrease continues to be accompanied by larger decreases in adolescent abortions compared with live births (*55*,*56*).

The findings in this report indicate that the number, rate, and ratio of reported abortions have declined across all race/ethnicity groups but that well-documented disparities persist (*3*,*4*,*15*–*20*). Comparatively high abortion rates and ratios among non-Hispanic black women have been attributed to higher unintended pregnancy rates and a greater percentage of unintended pregnancies ending in abortion (*57*,*58*). Data from certain reports suggest that differences in abortion indicators between non-Hispanic black women and women of other groups narrowed from 1994 to 2008 (*4*,*19*) but remained steady from 2008 to 2014 (*20*). Data in this report on abortions by race/ethnicity during 2007–2014 indicate large declines in abortion rates and ratios among non-Hispanic white and non-Hispanic black women, and differences between the two groups narrowed in the most recent period from 2010 to 2014. Higher abortion rates among Hispanic compared with non-Hispanic white women have been attributed to higher pregnancy rates, including intended and unintended pregnancies, among Hispanic women (*57*,*58*). However, abortion ratios for these two groups have been more comparable: Hispanic women have had a slightly higher percentage of pregnancies that are unintended but are no more likely than non-Hispanic white women to end unintended pregnancies in abortion (*57*,*58*). Data in this report on abortions by race/ethnicity during 2007–2014 indicate large declines in abortion rates for both non-Hispanic white and Hispanic women, and differences between these two groups narrowed from 2010 to 2014.

The findings in this report indicate the majority of women obtaining abortions do so early in gestation (≤8 weeks), when the risks for complications are lowest (*59*–*62*). Among the areas that reported gestational age data every year during 2005–2014, the percentage of abortions performed at ≤8 weeks’ gestation increased 2%. Moreover, among the areas that reported abortions at ≤13 weeks’ gestation by individual week, the distribution continued to shift toward earlier weeks of gestation, with the percentage of early abortions performed at ≤6 weeks’ gestation increasing 9% from 2005 to 2014. Nonetheless, the overall percentage of abortions performed at ≤13 weeks’ gestation was stable during 2005–2014, and many reports indicate that delays in obtaining an abortion are more common among certain groups of women (*63*–*65*). The findings in this report indicate that among women obtaining abortions, a smaller percentage of adolescents aged ≤19 years and non-Hispanic black women, compared with women in other age and race/ethnicity groups, obtain abortions at ≤8 weeks’ gestation. Because of the small but persistent percentage of women who obtain abortions at >13 weeks’ gestation, a better understanding is needed of how to address factors that cause delays in obtaining abortions (*63*,*65*–*68*).

The trend of obtaining abortions earlier in pregnancy has been facilitated by changes in abortion practices. Research conducted in the United States during the 1970s indicated that surgical abortion procedures performed at ≤6 weeks’ gestation, compared with 7–12 weeks’ gestation, were less likely to result in successful termination of the pregnancy (*69*). However, subsequent advances in technology (e.g., improved transvaginal ultrasonography and sensitive pregnancy tests) have allowed very early surgical abortions to be performed with completion rates exceeding 97% (*70*–*72*). Likewise, the development of early medical abortion regimens has allowed for abortions to be performed very early in gestation, with completion rates for regimens that combine mifepristone and misoprostol reaching 96%–98% (*73*). In 2014, 64.9% of all reported abortions were performed at ≤8 completed weeks’ gestation; thus, the women receiving these abortions were eligible for early medical abortion (a nonsurgical abortion at ≤8 weeks gestation) on the basis of gestational age; 33.3% of abortions at ≤8 weeks’ gestation and 22.5% of all abortions were reported as early medical abortions, with the proportion of all abortions reported as early medical abortion up from 10.7% in 2005. Moreover, in addition to abortions meeting the definition of early medical abortion, the percentage of medical abortions completed at 9 weeks’ gestation has increased in recent years (from 5.0%–6.8% during 2011–2013 to 7.7% in 2014). On the basis of evidence that early medical abortion is safe and effective beyond 63 days’ gestation (*42*), professional clinical practice guidelines were updated midyear in 2013 and 2014 to extend the gestational age eligibility for early medical abortion to 70 days (≤9 completed weeks) (*43*,*44*). In early 2016, FDA updated its approval for use of mifepristone for early medical abortions, extending the gestational age limit to 70 days (*45*). CDC will continue to monitor medical abortions at 9 weeks’ gestation.

The annual number of deaths related to legal induced abortion has fluctuated from year to year over the past 40 years. Because of this variability and the relatively small number of abortion-related deaths every year, national legal abortion case-fatality rates were calculated for consecutive 5-year periods during 1973–2007 and for a consecutive 6-year period during 2008–2013. The national legal induced abortion case-fatality rate for 2008–2013 was similar to the case-fatality rate for most of the preceding 5-year periods but was much lower than the case-fatality rate for the 5-year period (1973–1978) that immediately followed nationwide legalization of abortion in 1973.

## Limitations

The findings in this report are subject to at least four limitations. First, because reporting requirements are established by the individual reporting areas (*22*), the collection of data varies, and CDC is unable to obtain the total number of abortions performed in the United States. During the period covered by this report, the total annual number of abortions reported to CDC was consistently approximately 71% of the number recorded by the Guttmacher Institute (*74*), which uses numerous active follow-up techniques to increase the completeness of the data obtained through its periodic national census of abortion providers (*50*). Although most reporting areas collect and send abortion data to CDC, this information is submitted to CDC voluntarily. Consequently, during 2005–2014, four of the 52 reporting areas did not provide CDC data on a consistent annual basis, and for 2014, CDC did not obtain any information from California, Maryland, or New Hampshire.^¶¶¶¶^ In addition, whereas most reporting areas that send abortion data to CDC have laws requiring medical providers to submit a report for every abortion they perform to a central health agency, in New Jersey and the District of Columbia, medical providers submit this information voluntarily (*21*). As a result, the abortion numbers these areas report to CDC are incomplete.***** Moreover, even in states that legally require medical providers to submit a report for all the abortions they perform, enforcement of this requirement varies, and as a consequence several other reporting areas provide CDC with incomplete numbers.^†††††^

Second, because reporting requirements are established by the individual reporting areas, many states use reporting forms that do not follow the technical standards and guidance CDC developed in collaboration with the National Association of Public Health Statistics and Information Systems. Consequently, many reporting areas do not collect all the information CDC compiles on the characteristics of women obtaining abortions (e.g., maternal age, race, and ethnicity). Although missing demographic information can reduce the extent to which the statistics in this report represent all women in the United States, five nationally representative surveys of women obtaining abortions in 1987, 1994–1995, 2001–2002, 2008, and 2014 (*15*–*18*,*20*) have produced percentage distributions for most characteristics that are nearly identical to the percentage distributions reported by CDC. The exception is the percentage distribution of abortions by race/ethnicity. In particular, the percentage of abortions accounted for by non-Hispanic black women is higher in this report than the percentage determined on the basis of a recent nationally representative survey of women obtaining abortions (*20*). Differences might be attributable both to the high degree of imprecision for this variable that reduces the reliability of national survey results (*18*,*19*) and because the number of states that report to CDC by race/ethnicity continues to be somewhat lower than for other demographic variables. Importantly, some reporting areas that have not reported to CDC or have not reported cross-classified race/ethnicity data (e.g., California, Florida, and Illinois) have sufficiently large populations of minority women that the absence of data from these areas reduces the representativeness of CDC data.

Similar to the case for race/ethnicity, most states have only recently included medical abortion as a specific category on their reporting forms (*21*), which might reduce the accuracy of CDC’s estimates of the use of this method relative to other abortion methods, particularly for trend analysis. Furthermore, even in states with medical abortion on their reporting form, it is possible that this method is disproportionately undercounted. A higher percentage of the abortions provided in physician’s offices and smaller caseload facilities are early medical abortions (*8*,*50*,*75*), and these practices might be difficult to identify for reporting without active surveillance efforts (*75*). Nonetheless, a comparison of CDC data with mifepristone sales data^§§§§§^ suggests that CDC’s Abortion Surveillance System accurately describes the use of early medical abortion relative to other abortion methods in the United States (*76*). CDC data also might underreport use of early medical abortion with the definition of early medical abortion through eight completed weeks. Recent changes in clinical practice guidelines for the use of mifepristone and misoprostol indicate the safety and effectiveness past eight completed weeks of gestation. Although the use of medical abortion past this gestational age might have increased with these changes, this information is not captured in the current CDC definition.

Third, abortion data are compiled and reported to CDC by the central health agency of the reporting area in which the abortion was performed rather than the reporting area in which the woman lived. Thus, the available population (*30*–*39*) and birth data (*40*), which are organized by the states in which women live, differ in some cases from the population of women who undergo abortions in a given reporting area. This likely results in an overestimation of abortions for reporting areas in which a high percentage of abortions are obtained by out-of-state residents and an underestimation of abortions for states where residents frequently obtain abortions out of state. Limited abortion services, more stringent legal requirements for obtaining an abortion, or geographic proximity to services in another state might influence where women obtain abortion services. To adjust for these reporting biases, CDC attempts to categorize abortions by residence in addition to geographic occurrence. However, in 2014, CDC was unable to identify the reporting area, territory, or country of residence for 12.2% of reported abortions.

Finally, reporting areas provide CDC with aggregate numbers rather than individual-level records. Therefore, stratified analyses by socioeconomic status cannot be done.

## Public Health Implications

Ongoing surveillance of legal induced abortion is important for several reasons. First, abortion surveillance is needed to guide and evaluate the success of programs aimed at preventing unintended pregnancies. Although pregnancy intentions can be difficult to assess (*79*–*84*), abortion surveillance provides an important measure of pregnancies that are unwanted. Second, routine abortion surveillance is needed to assess trends in clinical practice patterns over time. Information in this report on the number of abortions performed through different methods (e.g., medical or surgical) and at different gestational ages provides the denominator data that are necessary for analyses of the relative safety of abortion practices. Finally, information on the number of pregnancies ending in abortion is needed in conjunction with data on births and fetal losses to more accurately estimate the number of pregnancies in the United States and determine rates for various outcomes of public health importance (e.g., adolescent pregnancies) (*52*,*85*).

According to the most recent national estimates from 2010, 18% of all pregnancies in the United States end in induced abortion (*86*). Multiple factors influence the incidence of abortion, including access to health care services and contraception (*87*–*89*); the availability of abortion providers (*8*,*9*,*50*,*90*–*92*); state regulations, such as mandatory waiting periods (*68*), parental involvement laws (*93*), and legal restrictions on abortion providers (*94*,*95*); increasing acceptance of nonmarital childbearing (*96*,*97*); shifts in the race/ethnicity composition of the U.S. population (*98*,*99*); and changes in the economy and the resulting impact on fertility preferences and use of contraception (*100*,*101*). However, despite the multiple influences on abortion, because unintended pregnancy precedes nearly all cases of abortions^¶¶¶¶¶^ efforts to reduce the incidence of abortion need to focus on helping women, men, and couples avoid pregnancies that they do not desire.

Providing women and men with the knowledge and resources necessary to make decisions about their sexual behavior and use of contraception can help them avoid unintended pregnancies. Recent data indicate that the proportion of pregnancies in the United States that were unintended decreased from 51% in 2008 to 45% during 2011–2013, after a slight increase from 2001 to 2008 (*51*). One factor that might have contributed to this decrease is the increase that occurred during the same period in the use of the most effective forms of reversible contraception, specifically intrauterine devices and hormonal implants, which are as effective as sterilization at preventing unintended pregnancy (*102*–*105*). Although use of intrauterine devices and implants has increased in recent years, use of these methods remains low in comparison with use of oral contraceptives and condoms, both of which are less effective at preventing pregnancy (*102*,*104*). The majority of reported abortions in 2014 were among women with a previous birth or previous induced abortion, events that also are opportunities for contraception counseling and initiation; contraception provision in the immediate postpartum and postabortion settings might increase access to methods considered safe for many women (*106*). Additionally, providing contraception for women at no cost can increase use of these methods and reduce abortion rates (*87*–*89*). Cost, as well as insufficient provider reimbursement and training, inadequate client-centered counseling or youth-friendly services, and low client awareness of available contraceptive methods are common barriers to accessing contraception (*107*–*109*). Removing these barriers can help improve contraceptive use, thereby reducing the number of unintended pregnancies (*107*,*110*) and consequently the number of abortions performed in the United States.

## Footnotes

* Hereafter, all abortions in this report are considered to be legally induced unless stated to be illegally induced.

† Data were not reported for ≥1 year by California (2005–2014), Louisiana (2005), Maryland (2007–2014), and New Hampshire (2005–2014).

§ Includes aspiration curettage, suction curettage, manual vacuum aspiration, menstrual extraction, sharp curettage, and dilation and evacuation procedures.

¶ CDC collects information only on the estimated number of weeks (not days) of gestation and acknowledges the conventional use of completed weeks of gestation to describe pregnancy duration. CDC’s category ≤8 weeks’ gestation thus includes abortions up through 8 weeks and 6 days. This closely corresponds to the performance measure for medical abortion proposed by the American College of Obstetricians and Gynecologists for the year encompassed by this surveillance report (*25*).

** The cutoff of ≤12 weeks has been selected on the basis of the implausibility of this procedure being performed at earlier gestational ages and on the basis of early research assessing the safety of intrauterine instillations starting at 13 weeks’ gestation (*27*,*28*)

†† Excludes four reporting areas that did not report or did not report by maternal residence (California, Florida, Maryland, and New Hampshire).

§§ States that did not report for ≥1 year since 1998 include Alaska (1998–2000), California (1998–2013), Louisiana (2005), Maryland (2007–2013), New Hampshire (1998–2013), Oklahoma (1998–1999), and West Virginia (2003–2004).

¶¶ Excludes California, Louisiana, Maryland, and New Hampshire.

*** Comparisons do not include Wyoming, which reported <20 abortions.

††† Comparisons by residence status do not include California, Florida, Maryland, or New Hampshire. Because these areas either did not report or did not report abortions by maternal residence, numbers are available only from other reporting areas where their residents obtained abortions, and as a consequence meaningful statistics cannot be reported.

§§§ Excludes Alabama, Arizona, California, Delaware, Florida, Georgia, Hawaii, Illinois, Kentucky, Louisiana, Maryland, Nevada, New Hampshire, Tennessee, Vermont, Wisconsin, and Wyoming.

¶¶¶ Excludes Alaska, Arizona, California, Connecticut, Delaware, District of Columbia, Florida, Hawaii, Illinois, Iowa, Kentucky, Louisiana, Maine, Maryland, Massachusetts, Michigan, Mississippi, Nebraska, Nevada, New Hampshire, New Mexico, New York State, North Carolina, North Dakota, Oklahoma, Pennsylvania, Rhode Island, South Carolina, Vermont, Washington, Wisconsin, and Wyoming.

**** Excludes Arkansas, California, Connecticut, District of Columbia, Florida, Georgia, Louisiana, Maryland, Massachusetts, Nebraska, Nevada, New Hampshire, New York State, North Carolina, Vermont, Washington, and Wyoming.

†††† Excludes California, Connecticut, Delaware, District of Columbia, Florida, Georgia, Illinois, Kentucky, Louisiana, Maryland, Massachusetts, New Hampshire, New Mexico, New York State, North Carolina, Pennsylvania, Rhode Island, Vermont, Wisconsin, and Wyoming.

§§§§ Excludes California, Connecticut, Delaware, District of Columbia, Florida, Georgia, Hawaii, Illinois, Louisiana, Maryland, Massachusetts, New Hampshire, New Mexico, New York State, North Carolina, Ohio, Vermont, Wisconsin, and Wyoming.

¶¶¶¶ In 2014, the most recent year for which the Guttmacher Institute has published data, abortions performed in California, Maryland, and New Hampshire accounted for 20% of the 926,200 abortions counted through the Guttmacher Institute’s national census of abortion providers (*50*).

***** In 2014, the abortion numbers that CDC obtained from the District of Columbia and New Jersey were 48% and 54%, respectively, of the abortion numbers that the Guttmacher Institute obtained for these areas through their national census of abortion providers (*50*).

††††† In 2014, the abortion numbers CDC obtained for Wyoming were <5% of the numbers obtained for this state by the Guttmacher Institute through their national census of abortion providers. CDC numbers for Hawaii were 57% of the Guttmacher Institute numbers. CDC numbers for Colorado, Connecticut, Nevada, New York (city and state combined), North Carolina, Oregon, Rhode Island, Tennessee, West Virginia, and Vermont were 74%–<90% of the Guttmacher Institute numbers. All other areas with legal reporting requirements that provided data to CDC obtained numbers that were at least 90% of the Guttmacher Institute numbers (*50*).

§§§§§ Because the sole distributor of mifepristone in the United States only sells this medication to licensed physicians, who must sign and return a prescriber’s agreement, sales data from this company are not limited by individual state reporting requirements or the difficulties of identifying smaller providers within the wider medical community.

¶¶¶¶¶ Recent estimates suggest that intended pregnancies account for <5% of all abortions (*29*).
